# Sigma-1 receptor-regulated efferocytosis by infiltrating circulating macrophages/microglial cells protects against neuronal impairments and promotes functional recovery in cerebral ischemic stroke

**DOI:** 10.7150/thno.77088

**Published:** 2023-01-01

**Authors:** Gufang Zhang, Qi Li, Weijie Tao, Pingping Qin, Jiali Chen, Huicui Yang, Jiaojiao Chen, Hua Liu, Qijun Dai, Xuechu Zhen

**Affiliations:** 1 Jiangsu Key Laboratory of Neuropsychiatric Diseases and Department of Pharmacology, College of Pharmaceutical Sciences, Soochow University, Suzhou, Jiangsu 215123, China.; 2 Department of Neurobiology, Hai'an Hospital of Traditional Chinese Medicine, Hai'an 226600, China.

**Keywords:** Ischemic stroke, Efferocytosis, Macrophage/microglia, Sigma-1 receptor, Rac1

## Abstract

**Background:** Efferocytosis of apoptotic neurons by macrophages is essential for the resolution of inflammation and for neuronal protection from secondary damage. It is known that alteration of the Sigma-1 receptor (Sig-1R) is involved in the pathological development of some neurological diseases, including ischemic stroke. The present study aimed to investigate whether and how Sig-1R regulates the phagocytic activity of macrophages/microglia and its significance in neuroprotection and neurological function in stroke.

**Methods:** The roles of Sig-1R in the efferocytosis activity of microglia/macrophages using bone marrow-derived macrophages (BMDMs) or using Sig-1R knockout mice subjected to transient middle artery occlusion (tMCAO)-induced stroke were investigated. The molecular mechanism of Sig-1R in the regulation of efferocytosis was also explored. Adoptive transfer of Sig-1R intact macrophages to recipient Sig-1R knockout mice with tMCAO was developed to observe its effect on apoptotic neuron clearance and stroke outcomes.

**Results:** Depletion of Sig-1R greatly impaired the phagocytic activity of macrophages/microglia, accordingly with worsened brain damage and neurological defects in Sig-1R knockout mice subjected to tMCAO. Adoptive transfer of Sig-1R intact bone marrow-derived macrophages (BMDMs) to Sig-1R knockout mice restored the clearance activity of dead/dying neurons, reduced infarct area and neuroinflammation, and improved long-term functional recovery after cerebral ischemia. Mechanistically, Sig-1R-mediated efferocytosis was dependent on Rac1 activation in macrophages, and a few key sites of Rac1 in its binding pocket responsible for the interaction with Sig-1R were identified.

**Conclusion:** Our data provide the first evidence of the pivotal role of Sig-1R in macrophage/microglia-mediated efferocytosis and elucidate a novel mechanism for the neuroprotection of Sig-1R in ischemic stroke.

## Introduction

Ischemic stroke is a devastating disease, and effective therapeutics against its neurological consequences remain a challenge 1. Accumulating evidence indicates that immune cells, especially brain-resident microglia and infiltrated circulating macrophages, are intimately involved in the pathological development of ischemic stroke 2, 3. At an early stage, blocking nutrition and oxygen supply in response to brain ischemia activates various cell death processes, including apoptosis, autophagy and necrosis 4. A large amount of damage-associated molecular patterns (DAMPs) released by all types of dead cells trigger inflammatory responses by activating microglial cells or macrophages 5. Excessive neuroinflammation further promotes neuronal and glial cell damage and impairs brain repair and functional recovery. Thus, cell death is an integral part of DAMP-induced inflammatory responses activated by ischemia and reperfusion.

Efferocytosis is a process by which dead cells or debris are rapidly and efficiently cleared by professional phagocytes such as macrophages, which helps to inhibit DAMP release and subsequent reactive inflammation 6, 7. Inflammation and tissue repair are impaired owing to failure to activate efferocytosis 8, 9. Defective efferocytosis leads to the accumulation of dead cells and debris, which exacerbates inflammation, ultimately inducing progressive brain damage with poor outcomes of ischemic stroke 10, 11. Brain resident microglia and infiltrated monocyte-derived macrophages are the essential elements of the innate immune responses during the acute phase following brain ischemic insult. Inflammatory M1 macrophages/microglia exacerbate disease by releasing detrimental mediators such as a cascade of proinflammatory cytokines, hydrolyzed proteases and peroxides, while resolving M2 macrophages favors the maintenance of tissue homeostasis by efficiently clearing dead/dying cells or debris and reducing the production of proinflammatory cytokines 12-14. Given the importance of efferocytosis, targeting efferocytosis regulation may be an effective pharmacological approach as a therapeutic strategy for neuronal damage-related diseases such as stroke.

Sigma-1 receptor (Sig-1R) is a chaperone protein that modulates multiple aspects of cellular functions, such as cell death, autophagy/apoptosis, neuronal differentiation, and inflammation. Alterations in Sig-1R functions have been implicated in a few neurological and psychiatric diseases, such as depression, addiction, schizophrenia and Parkinson's disease 15-19. In an ischemic stroke mouse model with permanent MCAO, Sig-1R knockout mice displayed significantly increased infarct volumes compared to littermate wild-type controls 20. The Sig-1R agonist Comp-AD was reported to produce a neuroprotective effect after transient middle cerebral artery occlusion in mice associated with ER stress 21. We have previously demonstrated that activating Sig-1R significantly suppresses LPS-induced proinflammatory cytokine release by microglia 18. In addition to regulating neuroinflammation, it was also shown that Sig-1R is involved in the regulation of autophagy and apoptosis of neuronal cells, including in stroke 22-24. It is known that macrophage-mediated efferocytosis plays an important role in cellular protection and inflammation regulation; however, little is known about how Sig-1R regulates efferocytosis and its contribution to the protective effects of Sig-1R in cerebral ischemia.

In the present study, we focused on the effects of Sig-1R on the phagocytic modulation of macrophages post ischemic stroke. We provide the first evidence that Sig-1R is an important regulator of efferocytosis in macrophages. We found that the phagocytic activity of apoptotic cells depended on the activation of Sig-1R. We further demonstrated that Sig-1R physically interacts with Rac1 and that Rac1 activity is critical for Sig-1R-stimulated efferocytosis in macrophages. Moreover, our data revealed that Sig-1R-mediated efferocytosis in circulating macrophages/microglia cells contributed largely to the protection both in brain tissue impairments and neurological functions in ischemic stroke.

## Materials and Methods

### Animals

C57BL/6 mice were purchased from Shanghai Laboratory Animal Center (SLAC, Shanghai, China), and sigma-1 receptor knockout (Sig-1R^-/-^) mice were purchased from Jackson Laboratory and bred on C57BL/6 mice. All animals were housed three to five per cage on a 12-hour light/12-hour dark cycle under specific pathogen-free conditions. Eight- to 12-week-old mice were used in all experiments, and all animal experiments were approved by the Institutional Animal Care and Use Committee at Soochow University.

### Ischemic stroke model

Ischemic stroke experiments were performed in male mice aged 8 to 12 weeks and weighing 20 to 23 g. The intraluminal filament technique was used to induce transient middle artery occlusion (tMCAO), as described previously 25. A silicone-coated nylon monofilament (Cinontech Corporation) was inserted into the middle cerebral artery via the right internal carotid artery and left *in situ* for 60 min. During the MCAO procedure, mice were kept on a heat pad to maintain the core temperature of 36.5° ± 5 °C. Mice that displayed neurologic deficits, including spastic limb tone, unbalanced walking or circling to the contralateral side, were used for the subsequent studies.

To evaluate the effect of Rac1 on Sig-1R-mediated efferocytosis in experimental stroke animals, mice were intravenously injected with the Rac1 inhibitor EHop-016 (i.v., 20 mg / kg) 1 h prior to intraperitoneal injection of the Sig-1R agonist PRE-084 (i.p., 10 mg / kg). At 5 d after reperfusion, mice were sacrificed, and brain tissues were collected and used for immunofluorescence analysis. EHop-016 (Selleck, Shanghai, China) was dissolved in a solution containing DMSO / 30% PEG / 1% Tween-80 26, and PRE-084 was dissolved in saline.

### Assessment of neurological deficits

Mouse neurological performance was measured with Longa's method with minor modifications in a blinded manner. Neurological performance is defined by the following: Score 0, no apparent neurological deficits; 1, body torsion and forelimb flexion; 2, right side weakness and thus decreased resistance to lateral push; 3, unidirectional circling behavior; 4, longitudinal spinning; and 5, no movement.

### Behavior assessment

#### Rotarod test

The motor ability of the mice was assessed by the latency on the rod as previously described 25. Briefly, mice were trained twice a day for 5 days and on the morning of the experimental day to reduce the variability between subjects. Mice were placed on the rotarod (UGO Basile, Italy), set to accelerate uniformly from 4 to 40 rpm within 5 min. All animals were tested three times with at least a 5 min break between tests, and the latency to fall off the rotarod was recorded. The mean value of the three trials was regarded as the latency. Motor function was examined before tMCAO and 7 and 14 days after tMCAO.

#### Corner test

The sensorimotor asymmetry of mice was calculated by the Corner test at 7 and 14 d later. Mice were placed between two boards with a 30° angle facing the corner. The mice will turn back to the open side while both sides of the vibrissae touch the corner. Normal mice turned right or left equally, while mice with tMCAO preferentially turned toward the nonimpaired side. The numbers of left and right turns were recorded from ten trials for each test.

#### Open-field test

The open field test was used to determine locomotor activity as previously described 15. Briefly, mice were placed in open field plastic cages (25 × 25 × 30 cm, JILIANG, Shanghai, China) equipped with computer-controlled photocells, and the total distance traveled and time spent in the center were quantified for 15 min according to the manufacturer's instructions.

### Histological analysis of mouse brains

Mice were euthanized 5 days after tMCAO, and brains were collected and dissected on ice and sectioned into 2 mm coronal sections followed by 2% TTC (2,3,5‐triphenyltetrazolium chloride) phosphate buffer incubation for 20 min at 37 °C in the dark as previously described 27. Normal brain tissue was stained and exhibited a red color, while the infarct area exhibited a white color. The percentage of the infarct areas to the total brain areas was calculated by morphometric analysis with Image J-Pro Plus.

### Bone marrow-derived macrophage preparation

Mouse bone marrow cells were flushed from femurs and tibias with PBS and then cultured in DMEM supplemented with 10% FBS, 1% penicillin-streptomycin, and 10 ng/ml macrophage colony-stimulating factor (M-CSF) for 6 days. Then, bone marrow-derived macrophages (BMDMs) were collected and prepared for subsequent stimulation and analysis.

### Brain cells and tissue preparation

Mice were transcardially perfused with 1 × phosphate-buffered saline (PBS) after anesthesia. For cell preparations, brain tissues were isolated with forceps, cut into 4 mm2 pieces with scissors, transferred to a homogenizer (Wheaton) with 3 ml of RPMI-1640 and ground with loose and tight pestles. Homogenates were filtered through a 70-μm cell strainer and centrifuged at 1500 rpm for 10 min. The pellet was resuspended and then subjected to Percoll (GE Healthcare) gradient centrifugation at 1000 × g without braking. Cells were collected from the interface between the 40% and 70% Percoll gradient, and single-cell suspensions were obtained by passing through 40-μm nylon mesh for flow cytometry (LSRFortessa cell analyzer, BD Biosciences) analysis.

For tissue preparation for histology and immunostaining, brains were fixed in 4% paraformaldehyde-buffered solution overnight at 4 °C, followed by 30% sucrose immersion for dehydration. Brain sections (20 μm thick) were used for immunostaining.

### Flow cytometry

Cells were resuspended and blocked with Fc block antibody (BD, Biosciences) for 20 min at room temperature. Then, antibody mix was added to the cells and stained for 30 min at room temperature in the dark. All samples were analyzed by using an LSRFortessa flow cytometer (BD Biosciences). Fluorochrome-conjugated mAbs specific for CD45, CD11b, CD86, Ly6G (1A8), and CD206 (2B10C42) were purchased from BioLegend. Doublets were excluded by gating out the population defined by forward scatter and side scatter in the flow cytometry in all the experiments.

### Immunofluorescence

Frozen brain sections (20 µm) were incubated with an Iba1 (Woko), CD16 / 32 (BD Bioscience), CD206 (Bio-Rad Laboratories), CD68 (Bio-Rad Laboratories), NeuN (Sigma-Aldrich) overnight at 4 °C and then an Alexa Fluor^TM^ 488 donkey anti-mouse antibody (1:500; Invitrogen), Alexa Fluor^TM^ 546 donkey anti-rabbit antibody (1:500; Invitrogen), or Alexa Fluor^TM^ 647 donkey anti-rat antibody (1:500; Invitrogen) for 1 h at room temperature. Sections were counterstained with DAPI for 10 min. For efferocytosis *in vivo*, apoptotic cells were labeled with TUNEL (Sigma Aldrich). At least two microscopic fields per section and three sections for each mouse were analyzed using a laser-scanning confocal fluorescence microscope (CarlZeiss, LSM710, Germany).

### Quantitative real-time PCR

Total mRNA was isolated from macrophages or brain tissues using TRIzol reagent (Invitrogen, USA) according to a previously reported protocol 28. cDNA was synthesized from mRNA with a random primer using an M-MLV reverse transcriptase kit (Takara, Japan) in a reaction of 1 µg of total RNA. mRNA expression levels were quantified by qRT-PCR using SYBR Green PCR master mix (Promega) and the StepOnePlus™ Real-time PCR System (ThermoFisher, USA) with specific primers (Table 1). The relative gene expression level was calculated relative to the GAPDH expression level.

### Rac1 activity assay

Rac1-GTP is an active Rac1 that specifically binds to the p21-binding domain of the Rac1 effector protein PAK1 29. After incubation with apoptotic cells, the level of active Rac1 in BMDMs was analyzed with an Active Rac1 Detection Kit (Cell Signaling Technology, USA). Briefly, BMDMs were lysed, and the lysate was incubated with GST-PAK1-PBD and GST beads for 4 h at 4 °C with gentle rotation. Then, the beads were collected and washed twice with lysis buffer, and the protein complex binding on the beads was eluted with 2×SDS loading buffer and denatured at 95-100 °C for 5 min. The levels of Rac1-GTP were measured by immunoblot assays and normalized to total Rac1 levels in cells.

### Co-IP assays

To determine whether Sig-1R interacts with Rac1, BMDMs were pretreated with Sig-1R agonist or antagonist for 30 min, and then apoptotic cells were added to BMDMs at a ratio of 5:1 (apoptotic cells: macrophages) for 1 h at 37 °C. After quenching nonphagocytosed cells, the macrophages were harvested and lysed with IP buffer (50 mM Tris-HCl (pH 7.4), 1% NP-40, 0.25% sodium deoxycholate, 150 mM NaCl, 1 mM EDTA, 1 mM PMSF) for 30 min at 4 °C, followed by 12,000 rpm centrifugation, and the supernatants were incubated with the appropriate antibodies at 4 °C overnight. Then, protein A / G magnetic beads (Selleck, Shanghai, China) were added to the samples and incubated for 3-4 h with rotation. Protein-bead complexes were washed 8 times to remove nonspecifically bound proteins. The beads were mixed with 2 × loading buffer, and proteins were separated and denatured in boiling water. To analyze the interaction site of Rac1 and Sig-1R, HEK293T cells transfected with Sig-1R and wild-type or mutant Myc-Rac1 plasmids were used for Co - IP assay. Co-IP assays were performed as described above, and samples were analyzed by SDS-PAGE followed by immunoblotting.

### Plasmid preparation and transfection

WT Myc-Rac1 plasmid was obtained from Addgene (37030), Rac1 mutants were generated with the Mut Express® Ⅱ Fast Mutagenesis Kit V2 (Vazyme, Nanjing, China). WT Sig-1R was cloned into pcDNA3.1 (Invitrogen) or pCDNA3.1-flag-C using HindIII and BamHI.

HEK 293T cells were transfected using a One-Step DNA Transfecter Kit (ENLIGHTEN Biotech, Shanghai, China). For cells grown to 80% confluence in 6-cm dishes, 4 μg of plasmid DNA and 4 µl of transfection reagent were added to 900 µl of serum-free DMEM. The mixture was incubated for 10 min at room temperature, added to wells containing 3 ml of fresh medium for 48 h and then used for Co-IP analysis.

### Dead cells preparation

HT-22 cells were seeded on 10-cm dishes at approximately 90% confluence, 5-chloromethylfluorescein diacetate (CMFDA; Molecular Probes) was added to the cells with serum-free DMEM at a 1 μM final concentration for 20 min at 37 °C, followed by two washes with PBS. Then, the cells were cultured with fresh culture medium with 10% FBS. Apoptosis of CMFDA-labeled HT-22 cells was achieved by H_2_O_2_ (0.8 mM) treatment under normal cell culture conditions for 20 h. Apoptotic cells were collected and centrifuged at 1500 rpm for 5 min, supernatants were discarded, and cells were resuspended in fresh culture medium. This method routinely yields > 70% annexin V^+^ late apoptotic/necrotic cells. For the efferocytosis assay, dead cells were added to macrophages at a ratio of 5:1 (dead cells: macrophages) for the indicated time at 37 °C.

### Efferocytosis assay

Approximately 5×10^5^ BMDMs were seeded in a 24-well plate, exposed to experimental stimuli and incubated with CMFDA-labeled apoptotic cells at a ratio of 1:5 at 37 °C for 30 min. The cells were collected, the proportion of BMDMs that exhibited increased fluorescence (corresponding to phagocytosis of fluorescent-labeled apoptotic cells) was determined by flow cytometry on a BD LSR Fortessa® flow cytometer (BD Biosciences) or by fluorescence microscopy on a confocal microscope (CarlZeiss, LSM710, Germany). For fluorescent observation, at least three images per well were captured for the efferocytosis assay.

### Adoptive transfer of macrophages

Bone marrow-derived macrophages (BMDMs) were prepared from either WT or Sig-1R knockout mice as described above. Before injection, BMDMs were transfected with lentivirus containing the GFP element. Cells were incubated at 37 °C in a 5% CO_2_ incubator for 16 h before the medium was changed to fresh complete medium. BMDMs were harvested 48 h after transfection and resuspended at 2 × 10^7^ cells/ml with PBS. WT or Sig-1R^-/-^ BMDMs were injected into Sig-1R^-/-^ mice subjected to tMCAO (i.v. 100 μl/mouse) through the tail vein 2 h after reperfusion. At day 5, mouse brain tissues were collected and subjected to immunofluorescence analysis.

### Statistical analysis

The statistical significance of differences between groups was analyzed by using GraphPad Prism 8 (GraphPad Software). The results are presented as the means ± SDs, and P values less than 0.05 were considered statistically significant. Unpaired Student's t test (two-tailed) or two-way ANOVA tests were performed to compare two or more groups after the data were confirmed to be normally distributed. Where applicable, post hoc multiple comparison tests were performed (Tukey for pairs and Bonferroni for multiple groups).

## Results

### Activating the sigma-1 (Sig-1R) receptor promotes macrophage efferocytosis

**Figure 1:**
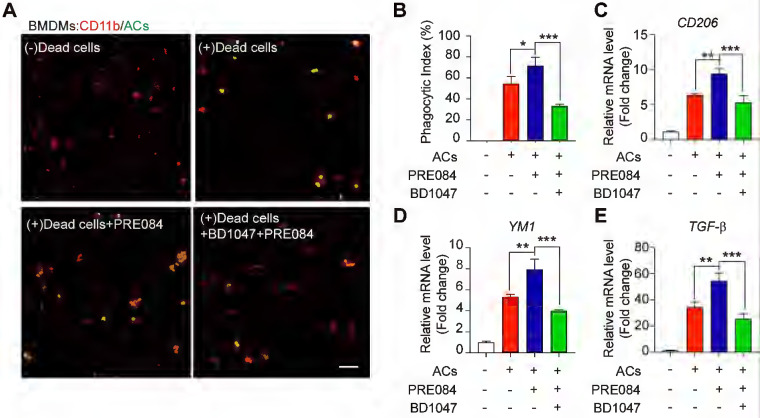
** Sig-1R activation promotes efferocytosis and M2 phenotype polarization of macrophages. (A)** Bone marrow -derived macrophages (BMDM) pre-treated with PRE-084 (10 μM) or BD1047 (10 μM) followed incubation with dead HT-22 neuronal cells which was prepared as described in Method for 30 min. Dead cells were labeled with CMFDA (Green), macrophages were marked with CD11b (Red). Scale bar: 50 μm. **(B)** Statistical analysis of phagocytotic index of macrophages.** (C)** mRNA level of *CD206*, **(D)** *YM1* and **(E)** *TGF-β* was determined 24 h in macrophages after incubation with dead cells. Data are presented as means ± SD, and were analyzed using one-way ANOVA followed by Dunnett's post-hoc tests, n = 3. *P < 0.05; **P < 0.01, ***P < 0.001.

To explore whether Sig-1R is involved in the regulation of efferocytosis, we investigated how Sig-1R activation regulates the clearance of dead cells in macrophages, which is a vital process for tissue injury and inflammation development after ischemic stroke. We employed HT-22 neurons as donors of dead cells (late apoptosis/necrosis) in which the cells were prelabeled with CMFDA (5-chloromethylfluorescein diacetate) and then incubated with hydrogen peroxide to induce up to 70% cell death (Figure S1). Bone marrow-derived macrophages (BMDMs) were preincubated with the Sig-1R agonist PRE-084 for 30 min and cocultured with dead/dying HT-22 cells at a 1:5 ratio as described in the figure legend. Confocal imaging analysis showed the enhanced uptake activity on dead cells by selective Sig-1R agonist PRE-084-treated macrophages compared with that of the control. Preincubation with the Sig-1R antagonist BD1047 abolished the effect of PRE-084 (Figure 1A-B), revealing a Sig-1R-mediated event. Consistent with the results, PRE-084 application to macrophages also enhanced the expression of biomarkers of the M2 phenotype, such as *Cd206, Ym1* and *Tgf-β1,* which was also blocked by BD1047 pretreatment after exposure to dead cells (Figure 1C-E). These results suggested that Sig-1R activation promoted macrophage uptake of dying/dead cells and facilitated macrophage switching to an anti-inflammatory phenotype.

### Engulfment of dead cells by macrophages/microglia is dependent on the sigma-1 receptor

**Figure 2:**
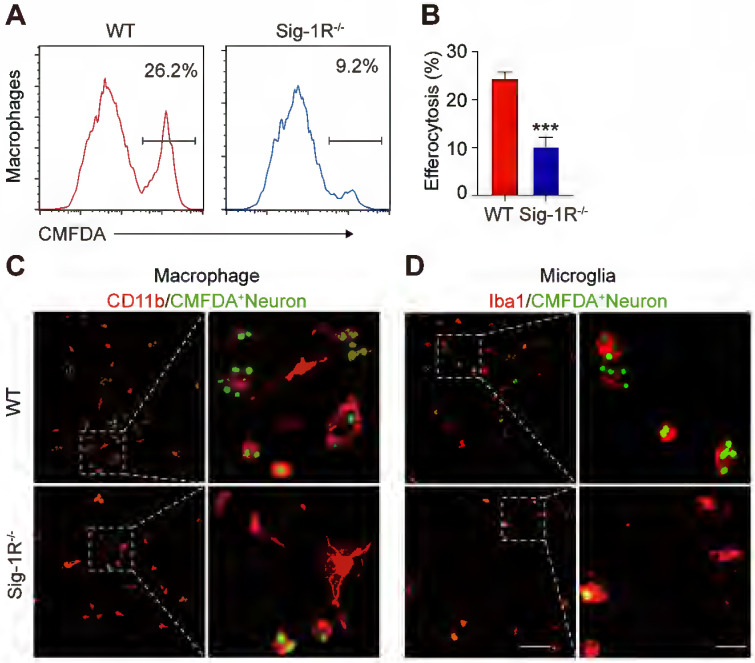
** Depletion of Sig-1R abrogates efferocytosis activity of macrophages.** Bone marrow derived macrophages (BMDMs) from either wildtype (WT) or Sig-1R knockout (Sig-1R^-/-^) mice were incubated with dead cells for 30 min. **(A)** Flow cytometry analysis of engulfed macrophage or microglia (CMFDA^+^ cells) was conducted as described. Presentative images of wildtype (WT) macrophages (Red line) and Sig-1R^-/-^ macrophages (blue lines) were exhibited. **(B)** Phagocytic index of macrophages was calculated as CMFDA^+^ cells among total cells.** (C)** WT or Sig-1R^-/-^ BMDMs were incubated with dead cells for 30 min at 1:5 ratio. Dead cells were labeled with CMFDA (Green) and macrophages were marked with CD11b (Red). Scale bar: 50 μm. **(D)** WT or Sig-1R^-/-^ primary cortical microglia were incubated with dead cells for 30 min at 1:5 ratio. Dead cells were labeled with CMFDA (Green) and macrophages were marked with Iba1 (Red). Scale bar: 50 μm. Data are presented as means ± SD and were analyzed using student *t* - test, n = 3. ***P < 0.001.

To further verify the effect of Sig-1R in regulating the engulfment of dead cells, we compared the phagocytic ability between WT and Sig-1R-deficient macrophages from Sig-1R knockout mice. Dying/dead HT-22 neuronal cells were labeled with CMFDA (green) and then cocultured with macrophages at a 1:5 (macrophages: dead cells) ratio as described. The percentage of engulfed dead cells (green) was analyzed by flow cytometry (Figure 2A). In relation to the fact that 25% of macrophages from WT mice were CMFDA positive, the proportion, however, was markedly decreased to approximately 8% in Sig-1R knockout macrophages (Figure 2B). Consistent with the results, we also observed a significant decrease in engulfed dead cells in Sig-1R knockout macrophages compared to WT macrophages (Figure 2C). M2 phenotypic markers such as *Ym1* and *CD206* were significantly higher (mRNA expression) in WT macrophages than in Sig-1R^-/-^ macrophages. The basal expression of the two markers was not altered between WT and Sig-1R^-/-^ macrophages (Figure S2). The protein expression of MerTK and CD36, both of which are critical molecules for recognizing dead cells, was not altered in WT and Sig-1R^-/-^ macrophages in response to dead cell treatment (Figure S3). In support of this hypothesis, we demonstrated that Sig-1R knockout disrupted phagocytic activity in primary microglia from Sig-1R knockout mice (Figure 2D). Interestingly, we noticed that macrophages appeared to have a stronger efferocytosis capacity than microglia, since we observed that CMFDA-positive macrophages engulfed more dead cells than microglial cells. In addition, we demonstrated that WT macrophages exhibited significantly stronger phagocytic activity toward E. Coli and zymosan bioparticles than Sig-1R^-/-^ macrophages (Figure S4). Our data thus clearly revealed that Sig-1R is involved in wide spectrum of efferocytosis by macrophages or microglia.

### Sigma-1 receptor knockout impairs clearance of apoptotic cells in response to cerebral ischemia

**Figure 3:**
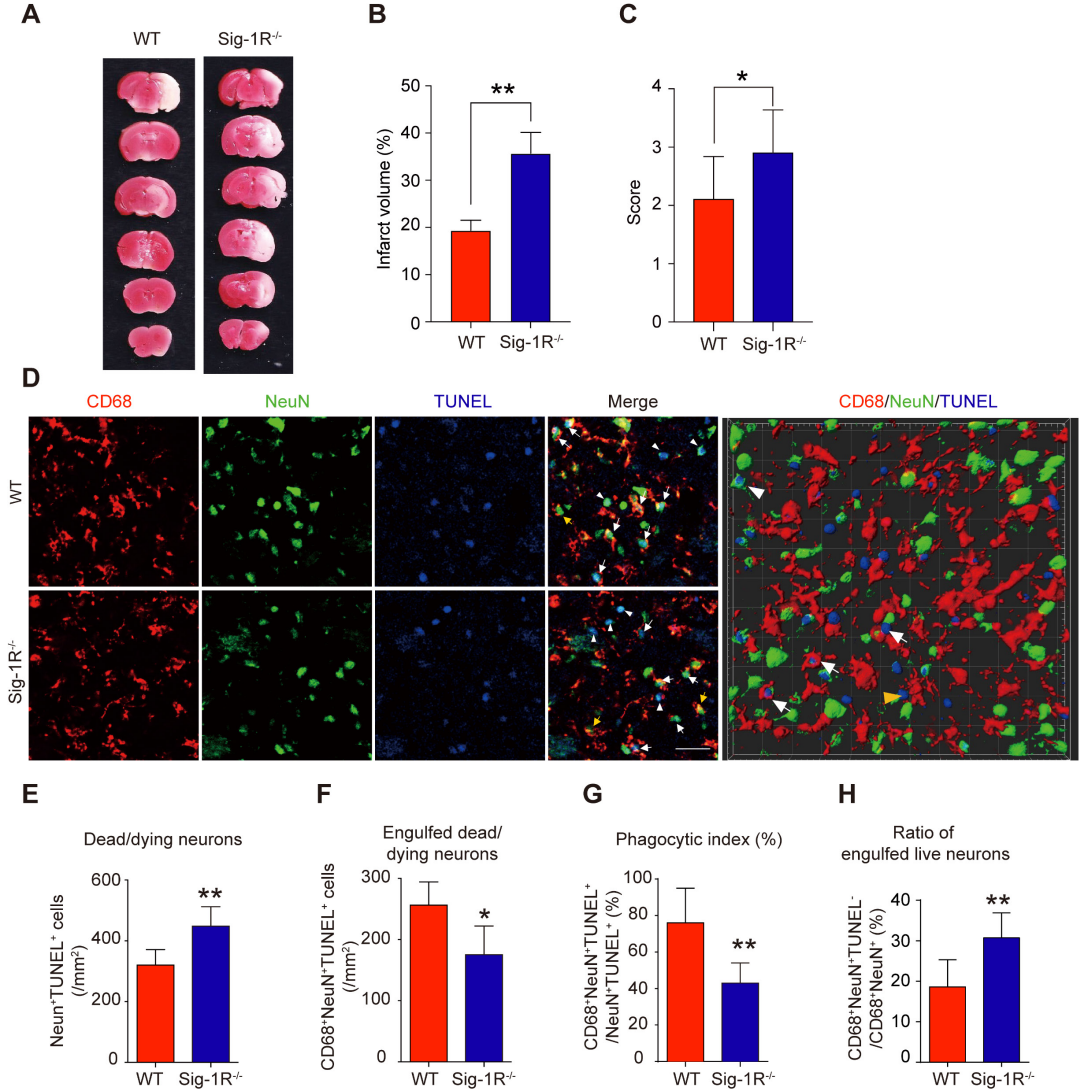
** Depletion of Sig-1R sensitized the brain injure and neurological defects companied with impaired phagocytic activity of macrophage/microglia.** WT or Sig-1R^-/-^ mice were subjected to tMCAO as described. Mice were monitored with neurological scores before sacrificed on day 5 after reperfusion. n = 7 mice / group. **(A)** Representative image of brain infarct area detected with TTC assay for each treatment.** (B)** Statistical analysis of infarct volume. **(C)** Neurological score for animals was assessed according to Material and Methods. **(D)** Representative images for CD68 (macrophage marker, Red) and NeuN (neuronal marker, Green) which was co-labeled with TUNEL (Blue) in infarct areas. Scale bar: 50μm. High-power 3-D image generated from D (right panel). White arrows indicate microglia / macrophages that engulfed dead / dying neurons (CD68^+^NeuN^+^TUNEL^+^). White arrowheads indicate dead / dying neurons that were not engulfed by microglia / macrophages (CD68^-^NeuN^+^TUNEL^+^). Yellow arrows indicate live neurons were engulfed by microglia / macrophages (CD68^+^NeuN^+^TUNEL^-^). Yellow arrowhead indicates a TUNEL^-^ neuron touched by an CD68^+^ cell (CD68^+^NeuN^+^TUNEL^-^). Scale bar: 50 μm. **(E)** The number of total dead / dying neurons in the ischemic striatum of mice. **(F)** The number of CD68^+^NeuN^+^TUNEL^+^ cells (microglia / macrophages with engulfed dead / dying neurons) in the ischemic striatum of mice. **(G)** Phagocytic index, as quantified the percentage of dead / dying neurons engulfed by microglia / macrophages ([number of CD68^+^NeuN^+^TUNEL^+^ cells / number of NeuN^+^TUNEL^+^cells] × 100%), in WT and Sig-1R^-/-^ mice brains. n = 6 mice per group. **(H)** Percentage of live neurons were engulfed by macrophages / microglia among all engulfed neurons ([number of CD68^+^NeuN^+^TUNEL^-^ cells / number of CD68^+^NeuN^+^ cells] × 100%) in WT and Sig-1R^-/-^ mice brains. n = 6 mice per group. Data are presented as means ± SD and were analyzed using student *t*-test. *P < 0.05; **P < 0.01.

To elucidate the role of Sig-1R in efferocytosis activity in ischemic stroke, we first explored the functional implication of Sig-1R in the acute phase in brain ischemia. Cerebral ischemia was induced by transient middle artery occlusion (tMCAO) and reperfusion in mice as described. As shown in Figure 3A&B, the brain infarct volume in Sig-1R^-/-^ mice was significantly larger than that in WT mice. In agreement with previous reports, we also confirmed that Sig-1R deletion exacerbated neurological deficits in response to ischemic stroke in tMCAO mice (Figure 3C). To observe the effect of Sig-1R on efferocytosis activity of macrophages/microglia *in vivo*, macrophages/microglia were labeled with CD68, which is predominantly expressed in late endosomes and lysosomes of macrophages 30; NeuN and TUNEL double-positive cells were marked as apoptotic neurons at the infarct striatum; CD68, NeuN and TUNEL triple-positive cells were apoptotic cells engulfed by macrophages/microglia. The high-power 3-D images clearly indicated phagocytic macrophages/microglia in the infarct striatum (Figure 3D). Consistent with the exacerbation of brain infarction, there were significantly more dead/dying neurons (NeuN^+^TUNEL^+^ cells) in the infarct striatum at 5 d after stroke in Sig-1R^-/-^ mice than in WT mice (Figure 3E). The number of macrophages/microglia that phagocytosed dead/dying cells (CD68^+^NeuN^+^TUNEL^+^) was significantly reduced in Sig-1R^-/-^mice (Figure 3F). In support of this hypothesis, the phagocytic index, which reveals the efferocytosis ability of macrophages/microglia, was markedly reduced in Sig-1R^-/-^ mice (Figure 3G). In addition to removing dead and dying neurons, microglia can also engulf live neurons 31. We also noted that a few live neurons (NeuN^+^TUNEL^-^) were engulfed by microglia/macrophages among all CD68 and NeuN double-positive cells (yellow arrow in Figure 3D). Interestingly, Sig-1R knockout mice exhibited a markedly higher percentage of engulfed live neurons in microglia/macrophages than WT mice (Figure 3H), and whether Sig-1R knockout contributes to exacerbated impairment in Sig-1R^-/-^ mice in response to ischemic stroke remains unclear. Taken together, these results suggest that Sig-1R plays a critical role in the regulation of the phagocyte function of macrophages/microglia that may contribute to brain injury following ischemic insult.

### Sigma-1 receptor deletion exacerbates inflammation after brain ischemia

**Figure 4:**
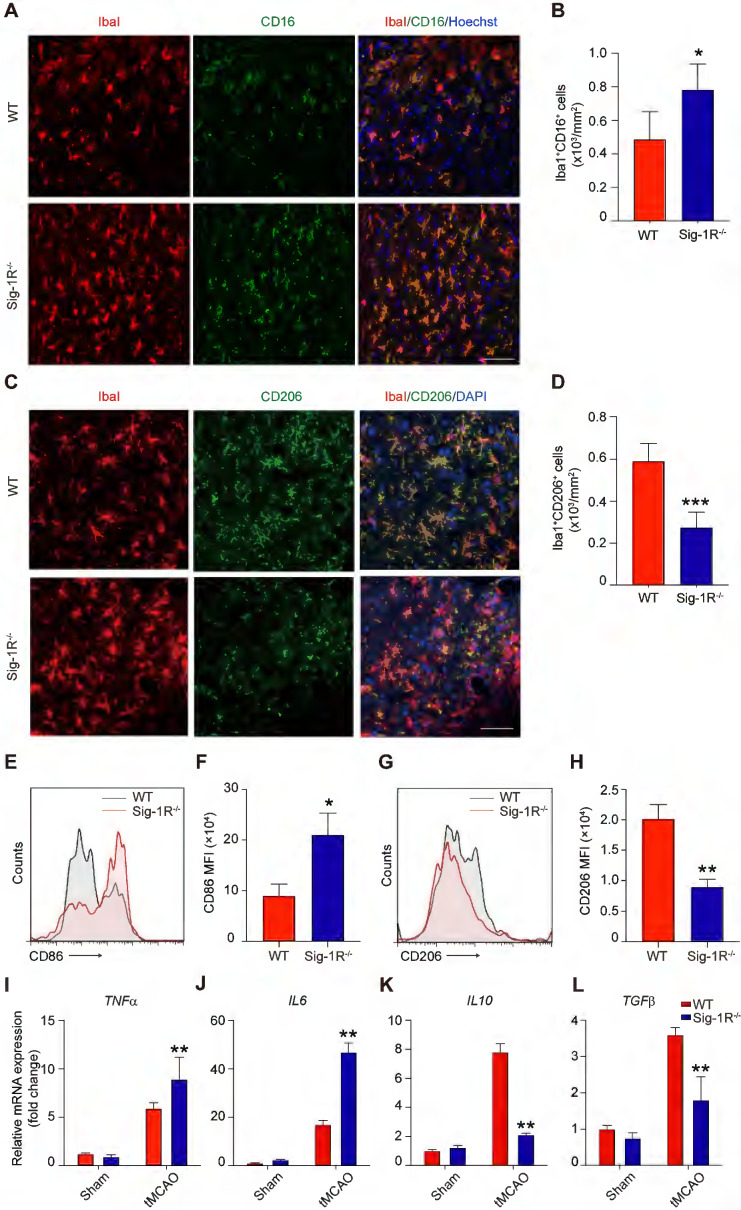
** Sig-1R deletion promotes macrophages/microglia activation and its tMCAO.** Brains of WT or Sig-1R^-/-^ mice were subjected to tMCAO as described, and brains were collected on day 5 for further experiments.** (A)** Representative images of microglia/macrophage marker Iba1 (Red) and inflammatory phenotype marker CD16 (Green) double-staining. Scale bar: 50 μm. **(B)** Quantification of Iba1^+^CD16^+^ proinflammatory macrophages/microglia in ischemic areas. n = 5 mice per group. **(C)** Representative images of Iba1 (Red) and CD206 (Green) double staining. Scale bar: 50 μm. **(D)** Quantification of Iba1^+^ / CD206^+^ macrophages/microglia in ischemic areas. n = 5 mice per group. Data are presented as means ± SD and were analyzed using student* t* -test. *P < 0.05; ***P < 0.001. **(E)** Flow cytometry analysis of CD86 expression in CD45^+^CD11b^+^Ly6G^-^ macrophages in the brain, mean fluorescent intensity (MFI) was calculated **(F)**. **(G)** Flow cytometry analysis of CD206 level in CD45^+^CD11b^+^Ly6G^-^ macrophages in the brain, mean fluorescent intensity (MFI) was calculated** (H)**. mRNA expression of proinflammatory cytokines *TNF-α* **(I)** and* IL-6* **(J)**, resolving cytokines *IL-10* **(K)** and *TGF-β* **(L)** were measured with RT-PCR in the ipsilateral hemisphere. n = 5 mice per group. Data are presented as means ± SD and analyzed with one-way ANOVA followed by Dunnett's post-hoc tests. **P < 0.01.

The phenotypic shift of macrophages/microglia in the acute phase of brain ischemia is an essential factor in the outcomes after stroke. To understand the roles of Sig-1R knockout on the polarization of macrophages/microglia after stroke, we first compared the pro-inflammatory phenotypic marker CD16 (Figure 4A) and anti-inflammatory marker CD206 (Figure 4C) expression in macrophages/microglia from the ischemic striatum at 5 d after reperfusion between WT and Sig-1R knockout mice. As shown in Figure 4B, the number of ionized calcium binding adaptor molecule 1 (Iba1) and CD16 double-positive cells was significantly higher in Sig-1R knockout mice. In contrast, the number of Iba1 and CD206 double-positive cells was reduced (Figure 4D). To further confirm the role of Sig-1R in the phenotypic polarization of infiltrated circulating macrophages after stroke, ischemic brains from WT or Sig-1R knockout mice 5 d after tMCAO were assessed and subjected to flow cytometry analysis. CD45^high^CD11b^+^Ly6G^-^ macrophages (sorting strategy shown in Figure S5) displayed a stronger MFI of the proinflammatory marker CD86 (Figure 4E-F) and a weaker MFI of the anti-inflammatory marker CD206 (Figure 4G-H) in Sig-1R knockout mice than in the same subset of macrophages from WT mice subjected to tMCAO.

Consistent with the phenotypic switch to a proinflammatory phenotype, Sig-1R deficiency led to augmented local inflammation. The mRNA expression of proinflammatory cytokines, including *TNF-α* (Figure 4I) and *IL-6* (Figure 4J), was significantly elevated at 5 d after stroke in Sig-1R^-/-^ mice. In contrast, the mRNA expression of the anti-inflammatory cytokines *IL-10* (Figure 4K) and *TGF-β1* (Figure 4L) was significantly decreased in Sig-1R ^-/-^ mice compared with WT mice. Taken together, these results indicated that Sig-1R defects promoted macrophage polarization into the M1 type and infiltration into the ischemic area, consequently sensitizing the neuroinflammation.

### Transferring intact Sig-1R macrophages rescues the acute neurological defect in Sig-1R knockout mice with tCAMO

**Figure 5:**
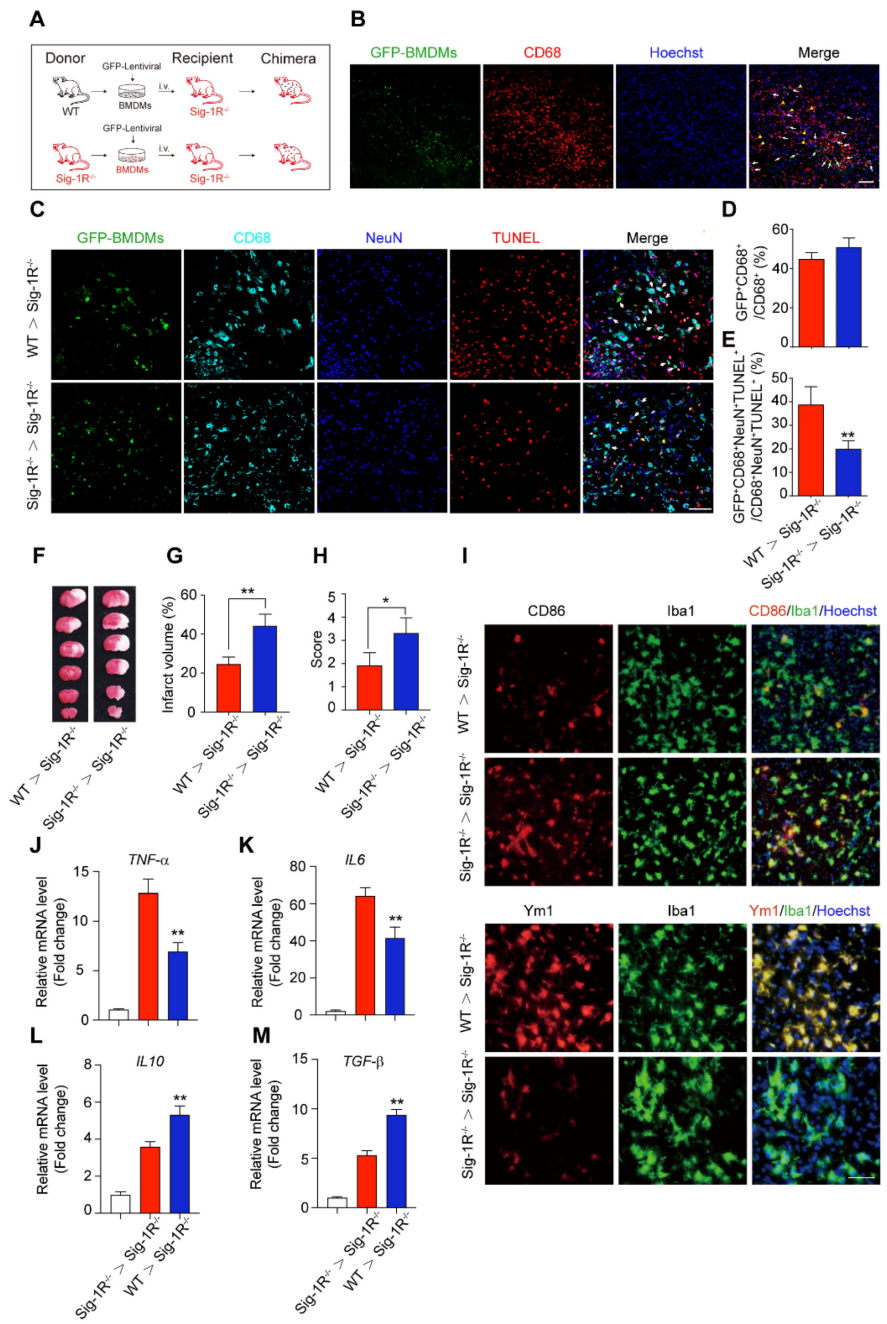
** Circulating macrophages attribute to Sig-1R-afforded protection against ischemic stroke. (A)** Age- and weight-matched Sig-1R^-/-^ mice as recipients were subjected tMCAO, BMDMs were prepared as depicted from either WT or Sig-1R^-/-^ mice. Chimeric mice were constructed by transferring WT or Sig-1R^-/-^ macrophages through tail vein injection to recipient Sig-1R^-/-^ mice immediately after reperfusion. **(B)** Reprehensive images indicating GFP^+^ BMDMs were accumulated at the brain infarct area, scale bar: 50 μm. **(C)** Reprehensive images depicting transferring BMDMs effectively phagocyte apoptotic neurons (white arrow, GFP^+^CD68^+^NeuN^+^TUNEL^+^), scale bar: 50 μm. **(D)** Percentage of transferred BMDMs (GFP^+^CD68^+^) among all CD68^+^ microglia/macrophages. (n = 5 mice per group) **(E)** Percentage of transferred BMDMs contribute to the efferocytosis among all the effectively phagocytes (GFP^+^CD68^+^NeuN^+^TUNEL^+^ / CD68^+^NeuN^+^TUNEL^+^). (n = 5 mice per group). **P < 0.01, one-way ANOVA.** (F)** Representative image of infarct area of for each treatment on day 5 after tMCAO. **(G)** The statistical analysis of infarction area and **(H)** neuronal score in each group was performed on day 5 after tMCAO. n = 7 mice / group.** (I)** Brains were collected from recipient mice and microglia/macrophage phenotypes were analyzed. Representative images for Iba1 (Green) and CD86 (Red) double staining and Iba1 with Ym1 (Red) double staining. Scale bar: 50 μm. mRNA expression of proinflammatory cytokines *TNF-α*** (J)** and *IL-6* **(K)**, *IL-10* **(L)** and *TGF-β* **(M)** were measured with RT-PCR in the ipsilateral hemisphere. n = 5 mice / group. **P < 0.01, data were analyzed with one-way ANOVA.

It is known that circulating macrophages infiltrate injured brain regions as early as 3 hours after reperfusion 32. These macrophages effectively clear dead cells or debris, which is critical for the pathological development and outcomes of ischemic stroke 10. To further elucidate the importance of Sig-1R in macrophages on neurological functions in response to cerebral ischemia, we transferred bone marrow-derived macrophages (BMDMs) obtained from either WT or Sig-1R^-/-^ mice to Sig-1R knockout ischemic mice 2 h after reperfusion (Figure 5A). BMDMs were transfected with lentivirus containing GFP elements prior to injection into recipient mice. Five days after injection, GFP-positive cells colocalized with CD68 accumulated in the infarct area (Figure 5B), and these BMDMs were able to engulf apoptotic cells (GFP^+^CD68^+^NeuN^+^TUNEL^+^) (Figure 5C). The basal number of GFP^+^CD68^+^ cells exhibited no significant difference between WT and Sig-1R^-/-^ mice (Figure 3D), while adoptive transduction of BMDMs from WT mice markedly enhanced efferocytosis activity compared with that of Sig-1R^-/-^ BMDMs (Figure 5E). Consistent with this observation, BMDMs transferred from WT mice greatly decreased the infarct volume of Sig-1R^-/-^ recipient mice, as did neurological functional impairment. In contrast, BMDMs transferred from Sig-1R-depleted mice did not produce any significant effects on those parameters (Figure 5F-H).

We also elucidated the roles of intact Sig-1R BMDMs in macrophage/microglia polarization in the ischemic area 5 days after ischemic insult. As indicated in Figure 5I, the expression of CD86 (red) in Iba1-positive (green) macrophages/microglia was significantly reduced in mice that accepted BMDMs from Sig-1R-intact WT mice but not Sig-1R-knockout mice. In contrast, the expression of Ym1 (red) in Iba1-positive (green) macrophages/microglia was significantly elevated (Figure 5I). Accordingly, we detected a significant decrease in *TNF-α* (Figure 5J) and *IL-6* (Figure 5K) mRNA expression. However, the expression of *IL-10* (Figure 5L) and *TGF-β* (Figure 5M) was significantly increased in mice treated with Sig-1R-intact BMDMs from WT mice compared to Sig-1R knockout mice. These data revealed that the transfer of sigma-1 receptor-intact macrophages was sufficient to polarize M1 macrophages into M2 phenotypes, leading to the inhibition of neuroinflammation. Taken together, the results indicated that Sig-1R-regulated macrophages/microglia play a critical role in acute brain damage and neurobiological functions in response to stroke.

**Figure 6:**
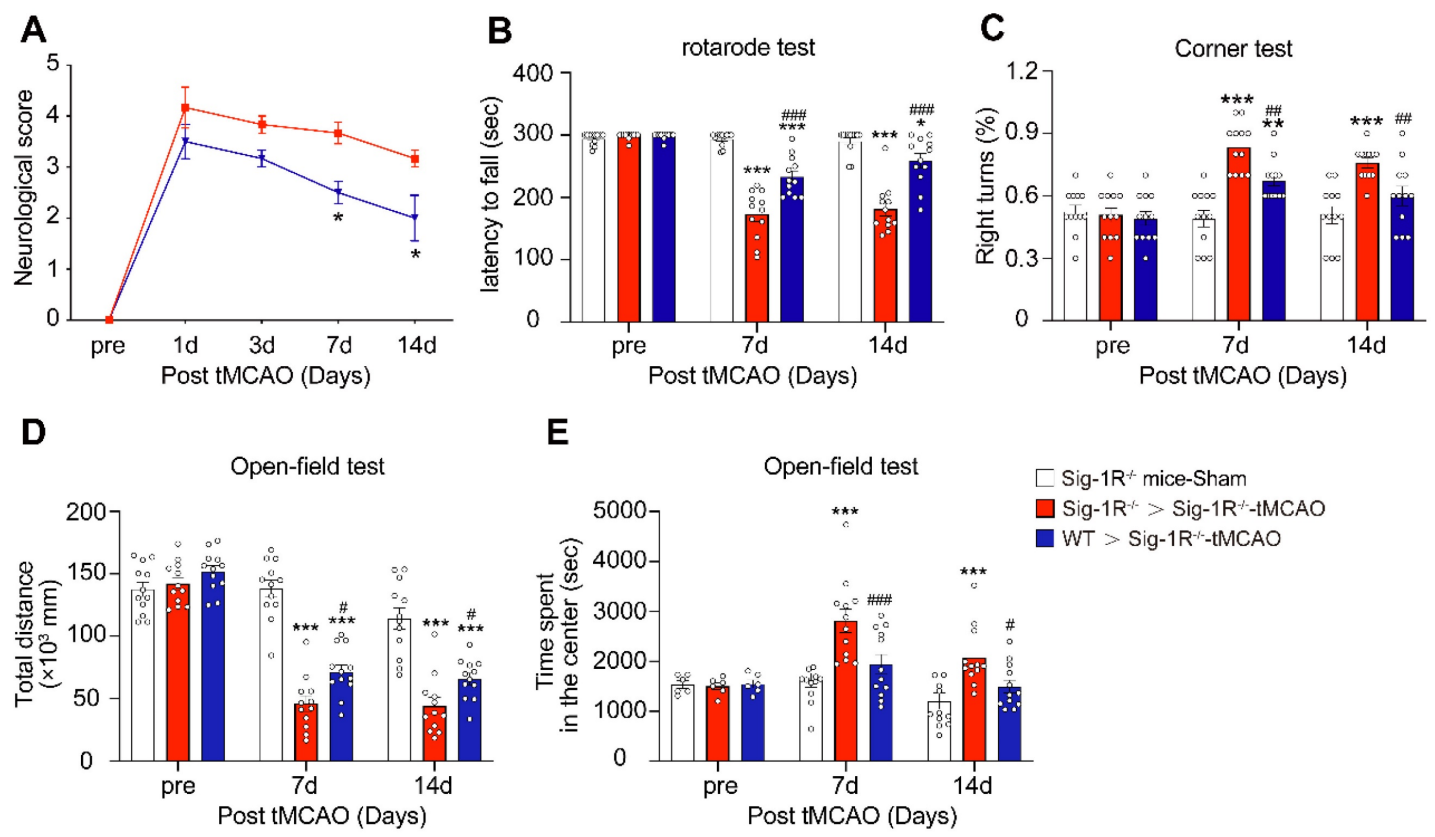
** Circulating WT macrophages alleviate neuronal deficits after ischemic stroke.** Sig-1R knockout mice were used as recipient mice to accepting WT or Sig-1R knockout BMDMs immediately after cerebral ischemia. Neurological function of recipient mice was observed each week. **(A)** Neurological scores were summarized (n = 6 mice per group).** (B)** The summary for rotarod test,** (C)** corner test and** (D and E)** open-field test were assessed to evaluate the motor and sensory function of recipient mice. n = 12 mice / group. Data are presented as means ± SEM. Two-way ANOVA followed by Dunnett's post-hoc tests were applied. *P < 0.05; **P < 0.01, ***P < 0.001 compared with sham group; #P < 0.05; ##P < 0.01, ###P < 0.001 compared with sham group.

To further evaluate the role of Sig-1R-modulated macrophages in long-term functional recovery after brain ischemia, neurological functions, including sensorimotor gating and cognitive ability, in recipients with BMDM transplantation from either WT or Sig-1R KO mice were assessed over a period of four weeks following tMCAO. Transferring macrophages from WT mice significantly ameliorated neurological deficits in Sig-1R KO mice compared to that of Sig-1R KO macrophage transplantation (Figure 6A), as did the rotarod test (Figure 6B) and corner test (Figure 6C). In addition, transferring macrophages from WT mice improved the general activity of animals and anxiety as measured by travel distance (Figure 6D) and the time spent in the center (Figure 6E), respectively, in the open-field test. This result indicated that transplantation with macrophages from WT mice to Sig-1R-depleted mice with tMCAO improved long-term neurological deficits. Taken together, the results indicated that macrophage Sig-1R-mediated protection is critical for decreasing brain damage and improving neurobiological functions after ischemic stroke.

### The sigma-1 receptor promotes efferocytosis through Rac1 signaling in macrophages

**Figure 7:**
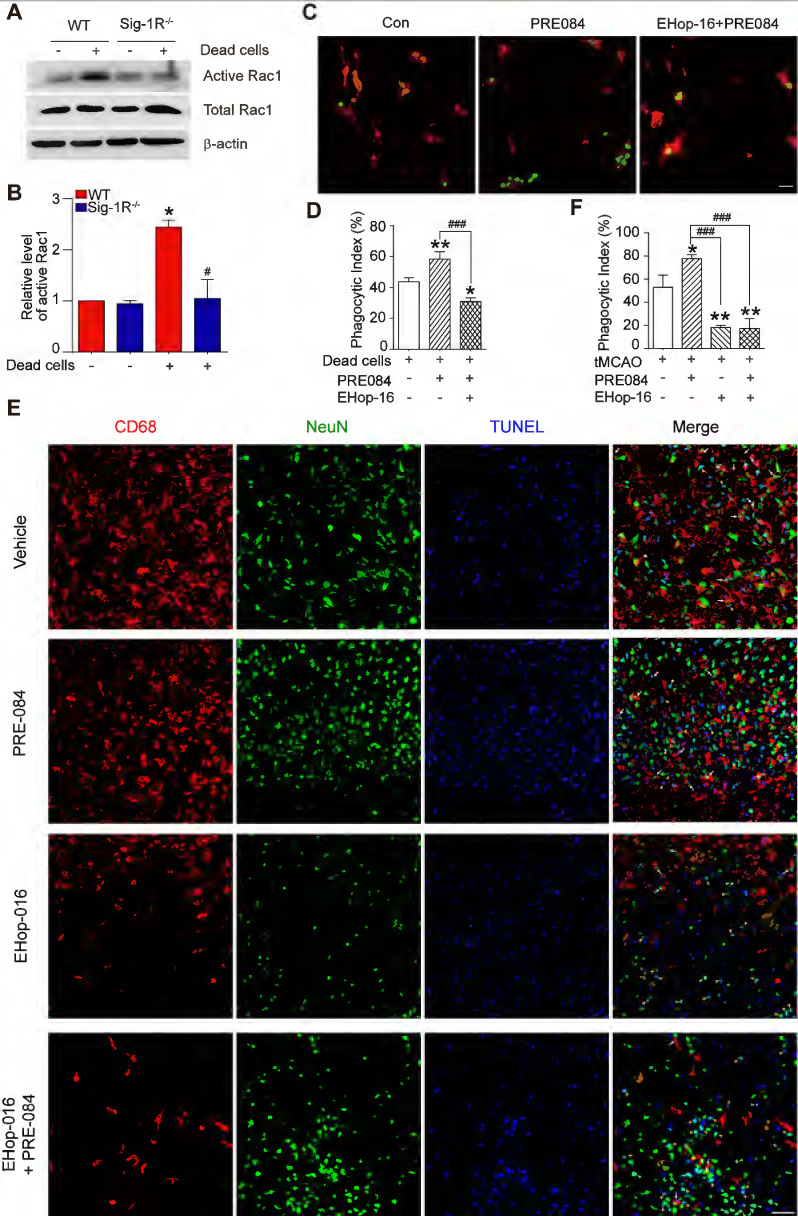
** Sig-1R regulate macrophage efferocytosis through Rac1. (A)** WT or Sig-1R^-/-^ BMDMs were incubated with dead cells for 1 h, Rac1 activity was determined.** (B)** Summary of Rac1 activity in WT and Sig-1R^-/-^ macrophages. n = 3, data are presented as means ± SD and analyzed with one-way ANOVA followed by Dunnett's post-hoc tests. *P < 0.05 versus WT without dead cells group. ^#^P < 0.05 versus WT with dead cells group. **(C)** Macrophages were pre-treated with Rac1 inhibitor EHop-016 (1 μM) for 30 min followed by Sig-1R agonist PRE084 (10 μM) prior to incubation with dead cells. Dead cells were labeled with CMFDA (Green) and macrophages were marked with CD11b (Red). Scale bar: 20 µM. **(D)** Phagocytotic index was determined as described. n = 3, data are presented as means ± SD and analyzed with one-way ANOVA followed by Dunnett's post-hoc tests. *P < 0.05, **P < 0.01 versus control group; ###P < 0.001 versus PRE-084-treated group.** (E)** Effects of Rac1 inhibitor EHop-016 on Sig-1R agonist PRE-084 induced efferocytosis in the mice brain infarct area after cerebral ischemia. Representative Images of CD68 (Red), NeuN (Green), and TUNEL (blue) triple-staining cells in each group. Scale bar: 50 μm. White arrows indicate apoptotic neurons were phagocyted by microglia/macrophages which resented as CD68^+^NeuN^+^TUNEL^+^ cells, yellow arrowheads indicate apoptotic neurons were not effectively phagocyted by microglia/macrophages which presented as CD68^-^NeuN^+^TUNEL^+^ cells. **(F)** Statistical analysis of phagocyte index in each group, which calculated as the percentage of CD68^+^NeuN^+^TUNEL^+^ among all NeuN^+^TUNEL^+^ cells. n = 5, data are presented as means ± SD and analyzed with one-way ANOVA followed by Dunnett's post-hoc tests. *P < 0.05, **P < 0.01 versus vehicle group; ###P < 0.001 versus PRE-084-treated group.

Thus far, we have discovered that Sig-1R plays a critical role in the regulation of efferocytosis. Depletion of Sig-1R resulted in impaired efferocytosis, which led to enhanced neuroinflammation and tissue damage, ultimately aggravating neurological defects. We next explored the potential mechanism for Sig-1R-regulated phagocytic activity of microglia/macrophages. It is known that the internalization of dead cells by phagocytes depends on actin polymerization, which is mediated by small GTPases, especially Rac1. Therefore, we first examined Rac1 activation in WT and Sig-1R^-/-^ macrophages. After incubation with dead cells, the activation of Rac1 was significantly decreased in Sig-1R^-/-^ macrophages compared to WT macrophages (Figure 7A-B). Furthermore, activation of Sig-1R by the specific agonist PRE-084 promoted phagocytic activity of macrophages, which was abrogated by EHop-016 (Figure 7C-D), a Rac1 inhibitor that has been proven to effectively inhibit Rac1 activity in the brain without affecting basal behavior through intraperitoneal injection 33. To further confirm the effect of EHop-016 on Sig-1R activation-induced clearance of dead neurons, ischemic mice were treated with EHop-016 (i.v., 20 mg/kg) via the tail vein 1 h before intraperitoneal administration of PRE-084 (i.p., 10 mg/kg). Five days post drug treatment, consistent with the *in vitro* results, PRE-084 treatment enhanced the clearance of apoptotic neurons (CD68^+^NeuN^+^TUNEL^+^) by microglia/macrophages compared to that of vehicle treatment. As expected, EHop-016 pretreatment markedly attenuated the Sig-1R activation-stimulated efferocytosis (Figure 7E-F). Interestingly, we observed that EHop-016 treatment also inhibited microglia/macrophage recruitment to the infarct area (Figure 7E). Taken together, these results indicated that Sig-1R-regulated efferocytosis of macrophages depends on Rac1 activation.

**Figure 8:**
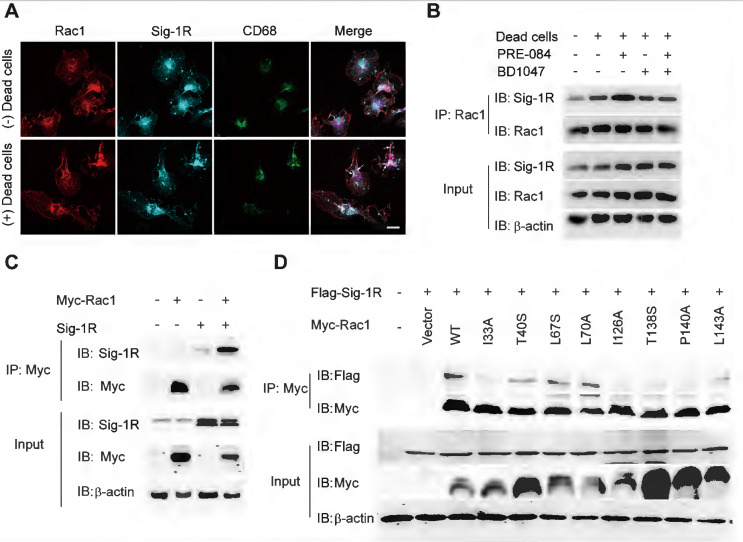
** Sig-1R activates and interacts with Rac1 to regulate macrophage efferocytosis. (A)** Macrophages were incubated with dead cells for 30 min before processed for immunostaining. Representative images of Rac1 (Red), Sig-1R (Cyan) and CD68 (Green) triple staining were shown from at least three repeated experiments with similar results. Scale bar: 20 μm. **(B)** Macrophages were pre-treated with BD1047 (10 μM) for 30 min before stimulation with PRE084 (10 μM) for 30 min. The cells were then incubated with dead cells for additional 60 min before collection for further assays. Co-immunoprecipitation (Co-IP) analysis for Rac1 and Sig-1R interaction was shown.** (C)** HEK293T cells were co-transfected with Myc-Rac1 and Sig-1R plasmids. the interaction between Rac1 and Sig-1R was monitored by co-immunoprecipitation (Co-IP) with anti-Myc beads, followed by SDS-PAGE separation and detected by respective antibody. **(D)** Single mutation of Rac1 plasmid was prepared as described in Method section, HEK293T cells were transfected with Flag-Sig-1R and wild type Myc-Rac1 or its single mutation, as indicated. After Co-IP with anti-Myc beads, the IP and input samples were separated by SDS-PAGE and probed with indicated antibodies.

Due to the chaperone nature of Sig-1R, we next investigated whether Sig-1R directly interacts with Rac1. As shown in Figure 8A, we detected enhanced colocalization between Rac1 and Sig-1R in macrophages in response to incubation with dead ells. This was further supported by the coimmunoprecipitation assays, which showed an elevated association between Rac1 and Sig-1R in macrophages that were preincubated with dead cells. Interestingly, we found that PRE-084 application enhanced the association, which was blocked by the Sig-1R-specific antagonist BD1047 (Figure 8B). We further confirmed the association *in vitro* using co-expressed Myc-tagged Rac1 and Sig-1R in HEK293T cells (Figure 8C). Interestingly, we found that after transfection with inactive Rac1 (T17N mutant), we detected a weak interaction between mutant Rac1 and Sig-1R, while constitutively active Rac1 (Q61 L mutant) enhanced its interaction with Sig-1R (Figure S6). To identify the structural basis for the interaction between Rac1 and Sig-1R, we used docking analysis to predict the interaction amino site (Table S1) based on the protein structure of Sig-1R and Rac1. Eight Rac1 residues with the highest interaction score were selected, and a single-mutated construct was generated and expressed in HEK293 cells, which included I33A, T40S (Switch I domain, GDP/GTP exchange), L67S, L70A (Switch Ⅱ domain, GDP/GTP exchange), I126A, T138S, P140A and L143A (Insert domain, membrane ruffling and actin organization). Coimmunoprecipitation assays showed that I33A, I126A, T138S or P140A mutations of Rac1 impaired the interaction with Sig-1R (Figure 8D).

## Discussion

It is well established that efferocytosis maintains tissue homeostasis through the removal of dead cells, which is believed to be a critical process in tissue damage and repair, inflammation modulation and neurological functions. The present study identified, for the first time, that Sig-1R plays a pivotal role in the regulation of macrophage/microglia efferocytosis, which contributes to receptor activation-promoted neuronal protection, tissue repair and neurological functional recovery in response to cerebral ischemic insults. The major discoveries of the present study are as follows: (1) Sig-1R is a critical regulator of efferocytosis activity in the clearance of dead cells by macrophages. Depletion of Sig-1R impaired the phagocytic ability of macrophages, consequently promoting neuroinflammation and tissue injury and sensitizing neurological defects in the ischemic brain. (2) Adoptive transfer of intact Sig-1R macrophages significantly attenuated tissue injury and neurological defects in Sig-1R knockdown mice subjected to ischemic stroke. (3) Sig-1R-mediated phagocytic activity depended on activation and interaction of the small GTPase protein Rac1.

Sig-1R is localized at mitochondria-associated membranes (MAMs) and acts as a chaperone protein. Upon activation with its agonist, such as PRE-084, the receptor dissociates from Bip and translocates to the plasma membrane or other sub-organelles to interact with other molecules to elicit multiple biological responses. PRE-084 and BD1047 is widely used as Sig-1R agonist and antagonist, respectively, which bind to the receptor to activate or inhibit the activation of the receptor. The neuroprotective effects of Sig-1R are generally believed to be associated with receptor activation-regulated autophagy, neuroinflammation and mitochondrial functions 17, 22. In addition, there were also studies shown that Sig-1R activation could induce GNDF/BDNF production 18, 34, 35, and blocking Sig-1R by BD-1047 markedly alleviated ethanol-induced neurotoxicity 36. Thus, targeting the Sig-1R receptor has been an important approach in drug discovery for a number of CNS diseases, such as AD, PD, depression, and stroke 19, 37. The present work, for the first time, revealed that Sig-1R is a critical regulator of efferocytosis that was associated with the ischemic brain damage, therefore, revealing a novel mechanism for Sig-1R-mediated neuroprotection.

Removing the dead/dying neurons or debris of apoptotic cells, i.e., efferocytosis is one of the key defense mechanisms for neuronal survival following neuronal damage, such as ischemic stroke, which is believed to be conducted by professional phagocytes, such as microglia, macrophages, neutrophils and dendritic cells. Effective clearance of dead cells in a timely manner is in favor of maintaining the brain homeostatic status by preventing DAMP release and subsequent excessive inflammation 38. Furthermore, engulfed dead cells promote microglia and macrophages to reprogram into the M2 phenotype, which contributes to inflammation resolution by producing anti-inflammatory cytokines, trophic factors and bioactive lipids and consequently accelerates tissue repair 6, 10, 39. The present data clearly demonstrated that Sig-1R is a key modulator of the phagocytic activity of macrophages, as supported by the following facts: activation of Sig-1R enhanced the phagocytic activity of macrophages on dead / dying cells, which was blocked by a Sig-1R antagonist; Sig-1R knockout macrophages greatly impaired its engulfment of dead cells (Figure 1A, Figure 2A - B). Thus, the present data provide the first evidence to reveal that Sig-1R plays a critical role in the regulation efferocytosis by macrophages or microglia. In support of this hypothesis, we found that activation of Sig-1R inhibited macrophage/microglial activation and promoted an M2 phenotype shift (Figure 1C-E), while depletion of the receptor sensitized the inflammation by stimulating the proinflammatory M1 phenotypes (Figure S2) 40. In addition, we found that depletion of Sig-1R also impaired phagocytic activity toward E. Coli and zymosan (Figure S4), indicating that loss of Sig-1R leads to a broader defect in phagocytosis.

We further investigated the functional roles of Sig-1R-regulated phagocytic activity in ischemic stroke. Dead astrocytes/neurons were observed within an hour after acute cerebral ischemia 10, DAMPs released from the dead cells induced microglial activation and peripheral macrophage infiltration into the injured region. It was reported that brain resident microglial cells are the immune cells that react the earliest to ischemic injury, and circulating macrophages enter the brain and accumulate in the infarct area within 24 h and peak at 3 days 10. These macrophages/microglia engulf dead neurons or apoptotic cells in response to “eat-me” signals 41-44. On the other hand, studies have shown that cell phagocytosis may also target living cells and lead to tissue damage 45. These results indicate that phagocytosis is a critical regulatory mechanism for neuronal damage and repair. Therefore, targeting phagocytic activity may represent a potential approach for ischemic stroke treatment 45. Indeed, it was shown that depletion of circulating monocytes during the first three days after stroke impairs long-term neuronal function recovery 46; transferred peripheral macrophages to mice reduced their infarct volume and ameliorated the accumulation of dead cells in the ischemic territory 47. Our data showed that there was a remarkable reduction in the phagocytic index at the infarct sites in Sig-1R knockout mice compared with WT mice subjected to tMCAO (Figure 3G), indicating an impairment of phagocytic activity in Sig-1R-deficient macrophages. It is also noted that we observed that live neurons were also engulfed by microglia/macrophages in the ischemic area, and Sig-1R^-/-^ mice appeared to have more live neurons engulfed than WT mice (Figure 3H). Whether it indicates an additional protective mechanism for Sig-1R remains to be studied. In agreement with previous reports 20, 21, we also confirmed that Sig-1R deletion exacerbated the infarction area and neurological deficits (Figure 3A-C) and promoted microglia/macrophage activation (Figure 4) in response to ischemic stroke in tMCAO mice. Consistently, transferring Sig-1R intact macrophages from WT mice improved acute and long-term neurological defects and brain damage in Sig-1R knockout mice with tMCAO (Figure 5 and 6), which may be attributed to the clearance of apoptotic neurons by transferred BMDMs (Figure 5B-C). Our data thus revealed that Sig-1R-regulated efferocytosis contributes to the protective effects of Sig-1R activation on brain injury and functional recovery in response to ischemic stroke. In addition, it is known that the polarization of microglia/macrophages plays a critical role in the process of tissue damage and functional recovery after ischemic stroke 10, 48. In agreement with previous reports 10, 49, 50, we also found that CD45^high^CD11b^-positive^ macrophages in the ischemic hemisphere of Sig-1R^-/-^ mice expressed significantly higher CD86 but lower CD206 than those of WT mice (Figure 4D), which exhibited a proinflammatory phenotype. The M1 phenotype, however, was switched to the M2 phenotype, while ischemic Sig-1R^-/-^ mice were adoptively transferred with intact sig-1R macrophages (Figure 5I). This confirms the important role of Sig-1R in macrophage/microglia polarization. This may also contribute to the beneficial effects of Sig-1R activation in brain damage and functional recovery after ischemic stroke 51, 52, which has been proved in our study that transfering WT BMDMs to Sig-1R^-/-^ mice subjected to tMCAO partly rescue impaired neurological functions (Figure 6). Another important question is what mechanisms underlie Sig-1R^-/-^-mediated macrophage efferocytosis. It is known that efferocytosis is a complex process, and any defect can cause alterations in macrophage engulfment and degradation 13. During the process of dead cells engulfment, GTPase, especially Rac1, is a crucial regulator in mediating actin remodeling, which results in the formation of apoptotic bodies 11. Interestingly, it was reported that Sig-1R physically interacts with Rac1 in mitochondria 53. We discovered that Sig-1R activation-mediated macrophage/microglia efferocytosis appears to depend on Rac1: (1) Rac1 activation was aborted in Sig-1R-depleted macrophages during efferocytosis (Figure 7A-B). (2) The Sig-1R activation-promoted phagocytic activity of macrophages was abolished by the Rac1-specific inhibitor EHop-016 *in vitro* and *in vivo* (Figure 7C and E). We further revealed that Rac1 activation promoted the interaction between Sig-1R and Rac1 (Figure 8, Figure S6). Furthermore, we mapped the structural basis for the interaction by conducting multiple mutations and identified that residues 33, 126, 138 and 140 of Rac1 appeared to be the key amino sites for the binding pocket (Figure 8D). However, whether these single alleles are decisive sites to mediate rac1-mediated efferocytosis in response to Sig-1R activation requires further investigation. On the other hand, how Rac1 interaction alters the translocation or functions of Sig-1R remains to be elucidated.

In summary, our study identifies Sig-1R as a pivotal molecule that mediates efferocytosis by macrophages, which contributes to inflammation resolution and functional recovery after cerebral ischemia. We also demonstrated that Sig-1R activation promotes phagocytic activity mainly through interaction with Rac1 in macrophages. The data revealed that modulation of Sig-1R/Rac1 signaling pathway-mediated phagocytic activity may be a potent therapeutic approach for ischemic stroke. It is also noted that although we provided sufficient evidence to demonstrate the importance of Sig-1R in the regulation of efferocytosis by macrophages/microglia, loss-of-function studies were performed using global Sig-1R knockout mice to develop macrophage-specific Sig-1R knockout mice, which may be a more precise approach to further elucidate the role of the receptor in phagocytic activity.

## Supplementary Material

Supplementary methods, figures, tables.

## Figures and Tables

**Table 1:** Primers used for qRT-PCR

Targets	Sequeces (5'-3') F: forward R: reverse
*GAPDH*	F: CATGGCCTCCAAGGAGTAAGA
	R: GAGGGAGATGCTCAGTGTTGG
*CD206*	F: AAACACAGATGACCCTTCCC
	R: GTTAGTGTACCGCACCCTCC
*Ym1*	F: GGGCATACCTTTATCCTGAG
	R: CCACTGAAGTCATCCATGTC
*TGF-β*	F: AGCTGCGCTTGCAGA GATTA
	R: TTCCGTCTCCTTGGTTCAGC
*TNF-α*	F: CATCTTCTCAAAATTCGAGTG ACAA
	R: TGGGAGTAGACAAGGTACAACCC
*IL-6*	F: TTCCATCCAGTTGCCTTCTT
	R: CAGAATTGCCATTGCACAAC
*IL-10*	F: GGTTGCCAAGCCTTATCGGA
	R: ACCTGCTCCACTGCCTTGCT

## References

[1] Powers WJ (2020). Acute ischemic stroke. N Engl J Med.

[2] Jian Z, Liu R, Zhu X, Smerin D, Zhong Y, Gu L (2019). The involvement and therapy target of immune cells after ischemic stroke. Front Immunol.

[3] Wang R, Liu Y, Ye Q, Hassan SH, Zhao J, Li S (2020). RNA sequencing reveals novel macrophage transcriptome favoring neurovascular plasticity after ischemic stroke. J Cereb Blood Flow Metab.

[4] Naito MG, Xu D, Amin P, Lee J, Wang H, Li W (2020). Sequential activation of necroptosis and apoptosis cooperates to mediate vascular and neural pathology in stroke. Proc Natl Acad Sci U S A.

[5] He T, Shang J, Gao C, Guan X, Chen Y, Zhu L (2021). A novel SIRT6 activator ameliorates neuroinflammation and ischemic brain injury via EZH2/FOXC1 axis. Acta Pharm Sin B.

[6] Boada-Romero E, Martinez J, Heckmann BL, Green DR (2020). The clearance of dead cells by efferocytosis. Nat Rev Mol Cell Biol.

[7] Doran AC, Yurdagul A Jr, Tabas I (2020). Efferocytosis in health and disease. Nat Rev Immunol.

[8] Doran AC, Ozcan L, Cai B, Zheng Z, Fredman G, Rymond CC (2017). CAMKIIgamma suppresses an efferocytosis pathway in macrophages and promotes atherosclerotic plaque necrosis. J Clin Invest.

[9] Kojima Y, Volkmer JP, McKenna K, Civelek M, Lusis AJ, Miller CL (2016). CD47-blocking antibodies restore phagocytosis and prevent atherosclerosis. Nature.

[10] Nakahashi-Oda C, Fujiyama S, Nakazawa Y, Kanemaru K, Wang Y, Lyu W (2021). CD300a blockade enhances efferocytosis by infiltrating myeloid cells and ameliorates neuronal deficit after ischemic stroke. Sci Immunol.

[11] Sharma M, Schlegel MP, Afonso MS, Brown EJ, Rahman K, Weinstock A (2020). Regulatory T cells license macrophage pro-resolving functions during atherosclerosis regression. Circ Res.

[12] Xu W, Roos A, Schlagwein N, Woltman AM, Daha MR, van Kooten C (2006). IL-10-producing macrophages preferentially clear early apoptotic cells. Blood.

[13] Yurdagul A Jr, Subramanian M, Wang X, Crown SB, Ilkayeva OR, Darville L (2020). Macrophage metabolism of apoptotic cell-derived arginine promotes continual efferocytosis and resolution of injury. Cell Metab.

[14] Liao S, Wu J, Liu R, Wang S, Luo J, Yang Y (2020). A novel compound DBZ ameliorates neuroinflammation in LPS-stimulated microglia and ischemic stroke rats: Role of Akt(Ser473)/GSK3beta(Ser9)-mediated Nrf2 activation. Redox Biol.

[15] Chen J, Li G, Qin P, Chen J, Ye N, Waddington JL (2022). Allosteric modulation of the sigma-1 receptor elicits antipsychotic-like effects. Schizophr Bull.

[16] Francardo V, Geva M, Bez F, Denis Q, Steiner L, Hayden MR (2019). Pridopidine induces functional neurorestoration via the sigma-1 receptor in a mouse model of parkinson's disease. Neurotherapeutics.

[17] Jia J, Cheng J, Wang C, Zhen X (2018). Sigma-1 receptor-modulated neuroinflammation in neurological diseases. Front Cell Neurosci.

[18] Wang Y, Guo L, Jiang HF, Zheng LT, Zhang A, Zhen XC (2016). Allosteric Modulation of sigma-1 receptors elicits rapid antidepressant activity. CNS Neurosci Ther.

[19] Ye N, Qin W, Tian S, Xu Q, Wold EA, Zhou J (2020). Small molecules selectively targeting sigma-1 receptor for the treatment of neurological diseases. J Med Chem.

[20] Rodriguez-Munoz M, Onetti Y, Cortes-Montero E, Garzon J, Sanchez-Blazquez P (2018). Cannabidiol enhances morphine antinociception, diminishes NMDA-mediated seizures and reduces stroke damage via the sigma-1 receptor. Mol Brain.

[21] Morihara R, Yamashita T, Liu X, Nakano Y, Fukui Y, Sato K (2018). Protective effect of a novel sigma-1 receptor agonist is associated with reduced endoplasmic reticulum stress in stroke male mice. J Neurosci Res.

[22] Wang M, Wan C, He T, Han C, Zhu K, Waddington JL (2021). Sigma-1 receptor regulates mitophagy in dopaminergic neurons and contributes to dopaminergic protection. Neuropharmacology.

[23] Zhang Y, Zhang X, Wei Q, Leng S, Li C, Han B (2020). Activation of sigma-1 receptor enhanced pericyte survival via the interplay between apoptosis and autophagy: Implications for blood-brain barrier integrity in stroke. Transl Stroke Res.

[24] Zhao X, Zhu L, Liu D, Chi T, Ji X, Liu P (2019). Sigma-1 receptor protects against endoplasmic reticulum stress-mediated apoptosis in mice with cerebral ischemia/reperfusion injury. Apoptosis.

[25] Jin Q, Cheng J, Liu Y, Wu J, Wang X, Wei S (2014). Improvement of functional recovery by chronic metformin treatment is associated with enhanced alternative activation of microglia/macrophages and increased angiogenesis and neurogenesis following experimental stroke. Brain Behav Immun.

[26] Lv L, Liu Y, Xie J, Wu Y, Zhao J, Li Q (2019). Interplay between alpha2-chimaerin and Rac1 activity determines dynamic maintenance of long-term memory. Nat Commun.

[27] Zhang G, Chen S, Jia J, Liu C, Wang W, Zhang H (2022). Development and evaluation of novel metformin derivative metformin threonate for brain ischemia treatment. Front Pharmacol.

[28] Zhang Y, Xu L, Zhang Y, Pan J, Wang PQ, Tian S (2022). Discovery of novel MIF inhibitors that attenuate microglial inflammatory activation by structures-based virtual screening and *in vitro* bioassays. Acta Pharmacol Sin.

[29] Higuchi M, Onishi K, Kikuchi C, Gotoh Y (2008). Scaffolding function of PAK in the PDK1-Akt pathway. Nat Cell Biol.

[30] da Silva RP, Gordon S (1999). Phagocytosis stimulates alternative glycosylation of macrosialin (mouse CD68), a macrophage-specific endosomal protein. Biochem J.

[31] Butler CA, Popescu AS, Kitchener EJA, Allendorf DH, Puigdellivol M, Brown GC (2021). Microglial phagocytosis of neurons in neurodegeneration, and its regulation. J Neurochem.

[32] Lee PT, Lievens JC, Wang SM, Chuang JY, Khalil B, Wu HE (2020). Sigma-1 receptor chaperones rescue nucleocytoplasmic transport deficit seen in cellular and Drosophila ALS/FTD models. Nat Commun.

[33] Liu Y, Du S, Lv L, Lei B, Shi W, Tang Y (2016). Hippocampal activation of rac1 regulates the forgetting of object recognition memory. Curr Biol.

[34] Penas C, Pascual-Font A, Mancuso R, Fores J, Casas C, Navarro X (2011). Sigma receptor agonist 2-(4-morpholinethyl)1 phenylcyclohexanecarboxylate (Pre084) increases GDNF and BiP expression and promotes neuroprotection after root avulsion injury. J Neurotrauma.

[35] Francardo V, Bez F, Wieloch T, Nissbrandt H, Ruscher K, Cenci MA (2014). Pharmacological stimulation of sigma-1 receptors has neurorestorative effects in experimental parkinsonism. Brain.

[36] Maurice T, Casalino M, Lacroix M, Romieu P (2003). Involvement of the sigma-1 receptor in the motivational effects of ethanol in mice. Pharmacol Biochem Behav.

[37] Guo CH, Cao T, Zheng LT, Waddington JL, Zhen XC (2020). Development and characterization of an inducible Dicer conditional knockout mouse model of Parkinson's disease: validation of the antiparkinsonian effects of a sigma-1 receptor agonist and dihydromyricetin. Acta Pharmacol Sin.

[38] Arandjelovic S, Ravichandran KS (2015). Phagocytosis of apoptotic cells in homeostasis. Nat Immunol.

[39] Zhang S, Weinberg S, DeBerge M, Gainullina A, Schipma M, Kinchen JM (2019). Efferocytosis fuels requirements of fatty acid oxidation and the electron transport chain to polarize macrophages for tissue repair. Cell Metab.

[40] Wu Z, Li L, Zheng LT, Xu Z, Guo L, Zhen X (2015). Allosteric modulation of sigma-1 receptors by SKF83959 inhibits microglia-mediated inflammation. J Neurochem.

[41] Brown GC (2021). Neuronal loss after stroke due to microglial phagocytosis of stressed neurons. Int J Mol Sci.

[42] Fu R, Shen Q, Xu P, Luo JJ, Tang Y (2014). Phagocytosis of microglia in the central nervous system diseases. Mol Neurobiol.

[43] Jia J, Yang L, Chen Y, Zheng L, Chen Y, Xu Y (2021). The role of microglial phagocytosis in ischemic stroke. Front Immunol.

[44] Schilling M, Besselmann M, Muller M, Strecker JK, Ringelstein EB, Kiefer R (2005). Predominant phagocytic activity of resident microglia over hematogenous macrophages following transient focal cerebral ischemia: an investigation using green fluorescent protein transgenic bone marrow chimeric mice. Exp Neurol.

[45] Neher JJ, Emmrich JV, Fricker M, Mander PK, Thery C, Brown GC (2013). Phagocytosis executes delayed neuronal death after focal brain ischemia. Proc Natl Acad Sci U S A.

[46] Wattananit S, Tornero D, Graubardt N, Memanishvili T, Monni E, Tatarishvili J (2016). Monocyte-derived macrophages contribute to spontaneous long-term functional recovery after stroke in mice. J Neurosci.

[47] Cai W, Dai X, Chen J, Zhao J, Xu M, Zhang L (2019). STAT6/Arg1 promotes microglia/macrophage efferocytosis and inflammation resolution in stroke mice. JCI Insight.

[48] Hu X, Li P, Guo Y, Wang H, Leak RK, Chen S (2012). microglia / macrophage polarization dynamics reveal novel mechanism of injury expansion after focal cerebral ischemia. Stroke.

[49] Ooi K, Hu L, Feng Y, Han C, Ren X, Qian X (2021). Sigma-1 receptor activation suppresses microglia M1 polarization via regulating endoplasmic reticulum-mitochondria contact and mitochondrial functions in stress-induced hypertension rats. Mol Neurobiol.

[50] Ruscher K, Shamloo M, Rickhag M, Ladunga I, Soriano L, Gisselsson L (2011). The sigma-1 receptor enhances brain plasticity and functional recovery after experimental stroke. Brain.

[51] Prasanth MI, Tencomnao T, Brimson JM (2021). The role of the sigma-1 receptor in neuroprotection: Comment on Nrf-2 as a therapeutic target in ischemic stroke. Expert Opin Ther Targets.

[52] Romeo G, Bonanno F, Wilson LL, Arena E, Modica MN, Pittala V (2021). Development of new benzylpiperazine derivatives as sigma-1 receptor ligands with *in vivo* antinociceptive and anti-allodynic effects. ACS Chem Neurosci.

[53] Natsvlishvili N, Goguadze N, Zhuravliova E, Mikeladze D (2015). Sigma-1 receptor directly interacts with Rac1-GTPase in the brain mitochondria. BMC Biochem.

