# Supplementary material for: Sigma-1 receptor-regulated efferocytosis by infiltrating circulating macrophages/microglial cells protects against neuronal impairments and promotes functional recovery in cerebral ischemic stroke
Source: Theranostics. 2023 Jan 1;13(2):543–59. doi: 10.7150/thno.77088 (PMC9830433; doi:10.7150/thno.77088)
Supplement: Supplementary file 1 — Supplementary methods, figures, tables. [file thnov13p0543s1.pdf]

Supplementary Methods

Phagocytosis of E.Coli or Zymosan bioparticles by BMDMs.

BMDMs were plated into 24-well cell imaging plate (Eppendorf, Hamburg, Germany) with 1×10^5 cells per well one day before phagocytosis assay. At day 2, replaced the culture medium with live cell imaging solution (Thermo), add pHrodo™ Green-labeled E.Coli (Thermo) or pHrodo™ Green-labeled Zymosan (Thermo) to the cell culture at 0.1 mg / ml and 0.05 mg / ml final concentration respectively, mix well and place the cells at 37°C for 60 min. Two microscopic fields per well and three wells for each genotype BMDMs were analyzed using a fluorescence microscope (OLYMPUS, IX73, Japan).

Supplementary Data

Figures

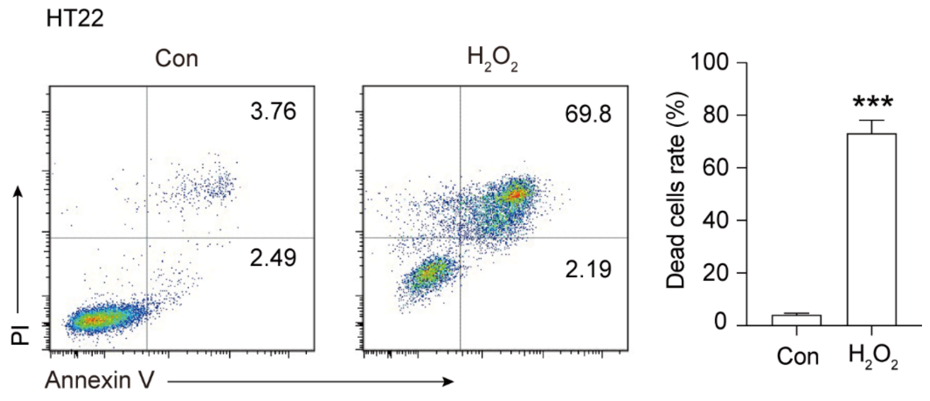

Figure S1. H_2O_2 induced apoptosis of HT-22 cells. Cells were incubated with H_2O_2 (800 μM) for 20 h and washed twice with PBS. After centrifuged at 1500 rpm for 5 min, supernatant was discarded and cell pellets were resuspended with binding buffer and stained with Annexin V and PI. Apoptosis population was analyzed with Flow cytometry. Data are presented as means ± SD and were analyzed using student *t* - test, n = 3. ***$P < 0.001$.

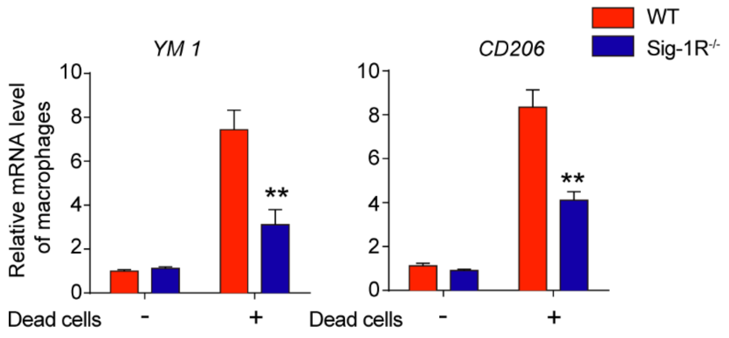

Figure S2. Sig-1R mediates M2 polarization in response to dead cells incubation. BMDMs from either WT or Sig-1R knockout mice were incubated with dead cells for 30 min. Dead cells were discarded, the cells were cultured for additional 24h before collection for assays. The mRNA expression of YM1 and CD206 were determined. Data are presented as means ± SD, and were analyzed using one-way ANOVA followed by Dunnett's post-hoc tests, n = 3. **P < 0.01 compared to WT macrophages.

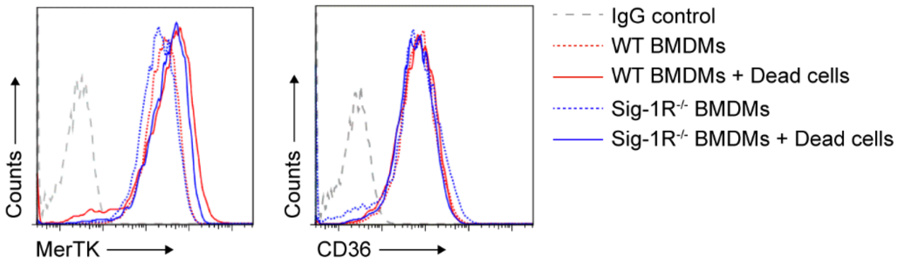

Figure S3. Expression of MerTK and CD36 in BMDMs were not altered by dead cells treatment. BMDMs from either WT or Sig-1R knockout mice were incubated with dead cells for 30 min, then dead cells were discarded and culture was changed with fresh complete medium for additional 24 h, BMDMs were collected and stained with MerTK or CD36 antibody followed by flow cytometry analysis.

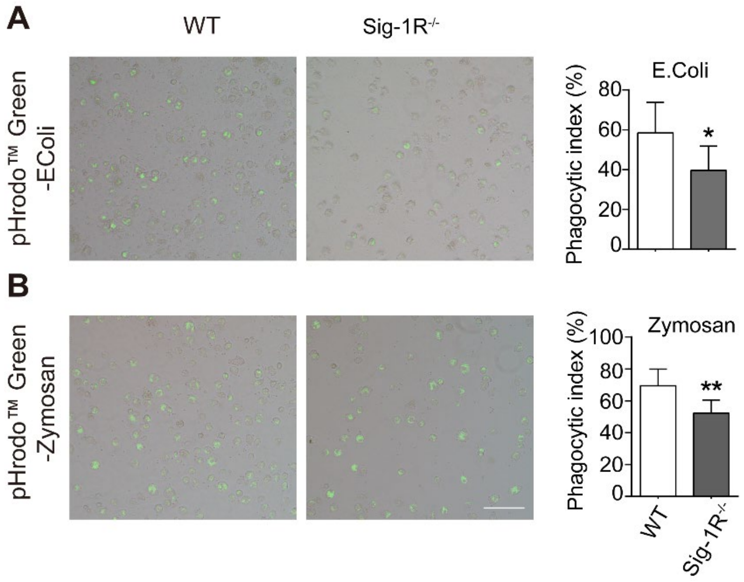

Figure S4. Sig-1R depletion impairs phagocytosis of E.Coli but not Zymosan of macrophages. BMDMs were incubated with either E.Coli or Zymosan for 60 min before subjected to staining by labeling with pHrodo™ Green dye for 60 min g. Representative images for E.Coli (A, left) or Zymosan (B, left). Scale bar: 100 μm. Phagocytosis index of E.Coli (A, right) or Zymosan (B, right) was displayed. One-way ANOVA followed by Dunnett's post-hoc tests, n = 3 wells / group, *$p < 0.05$ compared with WT macrophages.

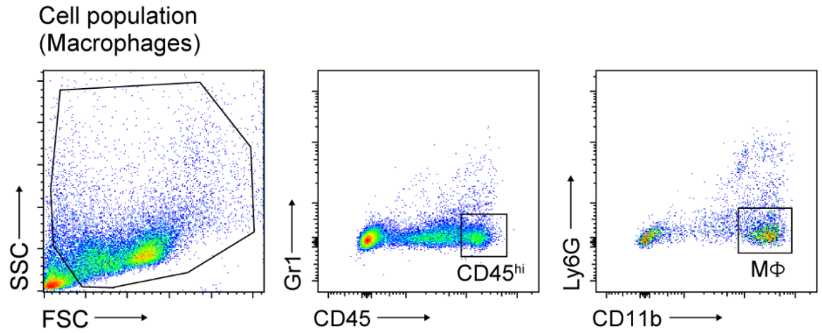

Figure S5. Flow cytometry gating strategy for brain CD11b^{+}Gr1^{-}Ly6G^{-} infiltrated macrophages. Mice were subjected tMCAO for 5 days, brains were collected and prepared for single cells depicted in Methods. Macrophages were identified as being CD45^{high}Gr1^{-}Ly6G^{-} cells.

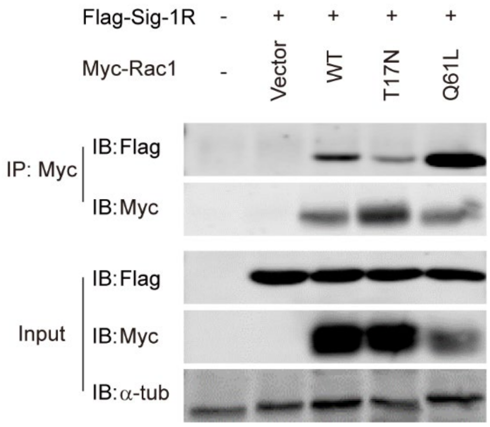

Figure S6. Activation of Rac1 promotes its interactionSig-1R. HEK293T cells were co-transfected with Flag-Sig-1R and wild type, inactive (T17N) or constitutively active Myc-Rac1 plasmid, respectively. The interaction between Rac1 and Sig-1R was evaluated by co-immunoprecipitation (Co-IP) with anti-Myc beads, followed by SDS-PAGE separation and detected by respective antibody. A representative image was shown. The experiments repeated at least three times with similar results.

Table S1. Three docking models were used for interaction sites of Sig-1R (5HK2) and Rac1 (3TH5) prediction with Discovery Studio.

Model 1 (Sig-1R-cyan; Rac1-magenta)

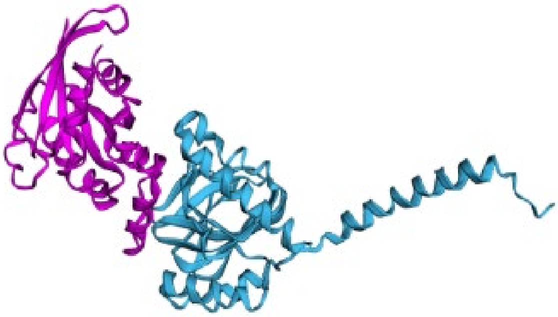

5HK2(Sig-1R)	Rank	Residues	Binding free energy	3TH5(Rac1)	Rank	Residues	Binding free energy
	1	A-MET-90	-4.68		1	B-PRO-140	-2.94
	2	A-LEU-111	-4.64		2	B-THR-138	-2.62
	3	A-PHE-83	-4.4		3	B-ILE-126	-2.23
	4	A-THR-193	-2.46		4	B-LYS-130	-1.66
	5	A-LEU-197	-1.89		5	B-THR-135	-1.65
	6	A-TYR-201	-1.48		6	B-ARG-163	-1.53
	7	A-TRP-81	-1.21		7	B-TYR-139	-1.1
	8	A-ALA-110	-1.02		8	B-GLN-141	-1.07

	9	A-TRP-169	-0.9		9	B-LYS-133	-0.99
	10	A-SER-113	-0.64		10	B-PRO-136	-0.91
	11	A-THR-198	-0.56		11	B-ILE-137	-0.91
	12	A-HIE-54	-0.53		12	B-ARG-120	-0.76
	13	A-ALA-92	-0.37		13	B-PRO-87	-0.61
	14	A-VAL-82	-0.27		14	B-ARG-174	-0.37
	15	A-PHE-196	-0.24		15	B-LYS-16	-0.35
	16	A-ASN-167	-0.22		16	B-ARG-94	-0.35
	17	A-GLY-91	-0.21		17	B-LYS-166	-0.35
	18	A-ALA-86	-0.09		18	B-LYS-96	-0.34
	19	A-TRP-89	-0.07		19	B-ARG-102	-0.34
	20	A-TRP-164	-0.07		20	B-ARG-68	-0.33
	21	A-PHE-58	-0.06		21	B-PHE-90	-0.33
	22	A-MET-93	-0.06		22	B-LYS-153	-0.3

	23	A-VAL-84	-0.05		23	B-LYS-5	-0.27
	24	A-LEU-56	-0.04		24	B-LYS-49	-0.27
	25	A-GLN-80	-0.04		25	B-ARG-66	-0.26

Model 2 (Sig-1R-cyan; Rac1-Green)

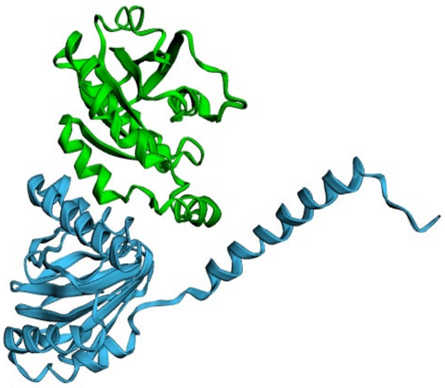

5HK2(Sig-1R)	Rank	Residues	Binding free energy	3TH5(Rac1)	Rank	Residues	Binding free energy
	1	A-LEU-203	-3.83		1	B-PRO-140	-3.79
	2	A-TRP-29	-3.54		2	B-LEU-143	-1.96
	3	A-VAL-26	-3.33		3	B-TYR-139	-1.93
	4	A-LEU-100	-2.75		4	B-ALA-144	-1.62
	5	A-PRO-179	-2.06		5	B-ILE-126	-1.34

	6	A-LEU-214	-2.05		6	B-LYS-123	-1.19
	7	A-LEU-30	-2.04		7	B-GLN-141	-1.12
	8	A-LEU-210	-1.98		8	B-THR-138	-0.99
	9	A-PHE-196	-1.96		9	B-LYS-133	-0.8
	10	A-TYR-206	-1.54		10	B-THR-135	-0.61
	11	A-TYR-217	-1.53		11	B-PRO-136	-0.4
	12	A-PHE-200	-1.5		12	B-ARG-174	-0.38
	13	A-LEU-182	-1.48		13	B-LYS-166	-0.3
	14	A-LEU-218	-1.2		14	B-LYS-49	-0.29
	15	A-VAL-25	-1.1		15	B-ILE-137	-0.29
	16	A-LEU-197	-1.04		16	B-ARG-102	-0.27
	17	A-ALA-207	-0.96		17	B-GLN-2	-0.25
	18	A-LEU-22	-0.86		18	B-LYS-16	-0.24
	19	A-TRP-121	-0.83		19	B-LYS-96	-0.24

	20	A-PHE-191	-0.72		20	B-LYS-5	-0.23
	21	A-LEU-186	-0.69		21	B-ARG-68	-0.23
	22	A-LEU-199	-0.56		22	B-ARG-66	-0.18
	23	A-ALA-183	-0.47		23	B-ARG-94	-0.18
	24	A-VAL-190	-0.43		24	B-LEU-129	-0.15
	25	A-TRP-27	-0.42		25	B-LYS-116	-0.14

Model 3 (Sig-1R-cyan; Rac1-turquoise)

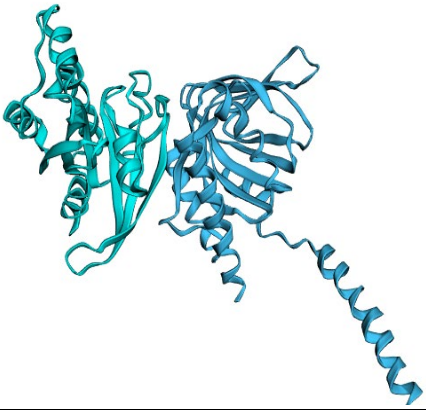

**5HK2(Sig-1R)**	**Rank**	**Residues**	**Binding free energy**	**3TH5(Rac1)**	**Rank**	**Residues**	**Binding free energy**
	1	A-TRP-81	-3.98		1	B-ILE-33	-4.17
	2	A-PHE-83	-3.19		2	B-LEU-70	-3.04

	3	A-LEU-197	-3.04		3	B-VAL-36	-2.89
	4	A-GLN-80	-2.72		4	B-TYR-40	-2.84
	5	A-LEU-111	-2.65		5	B-LEU-67	-2.58
	6	A-TYR-201	-1.73		6	B-PHE-37	-2.45
	7	A-GLU-77	-1.45		7	B-SER-41	-2.12
	8	A-TRP-169	-1.35		8	B-ILE-21	-1.79
	9	A-MET-90	-1.11		9	B-THR-25	-1.54
	10	A-ALA-110	-0.99		10	B-TYR-64	-1.32
	11	A-HIE-54	-0.89		11	B-ASN-39	-0.91
	12	A-ASN-167	-0.8		12	B-TRP-56	-0.89
	13	A-ALA-92	-0.71		13	B-PRO-29	-0.74
	14	A-VAL-82	-0.7		14	B-ASN-52	-0.53
	15	A-THR-198	-0.55		15	B-GLY-30	-0.5
	16	A-ASP-76	-0.52		16	B-ARG-102	-0.39

	17	A-PHE-200	-0.5		17	B-LYS-49	-0.38
	18	A-THR-193	-0.45		18	B-LYS-128	-0.36
	19	A-GLN-194	-0.31		19	B-ARG-174	-0.36
	20	A-LEU-56	-0.27		20	B-LYS-96	-0.35
	21	A-PHE-196	-0.25		21	B-LYS-116	-0.35
	22	A-PHE-58	-0.16		22	B-ARG-68	-0.34
	23	A-CYS-94	-0.15		23	B-LYS-132	-0.34
	24	A-ARG-114	-0.13		24	B-LYS-153	-0.33
	25	A-MET-93	-0.12		25	B-ARG-94	-0.32
